# Genome-wide association study for hereditary ataxia in the Parson Russell Terrier and DNA-testing for ataxia-associated mutations in the Parson and Jack Russell Terrier

**DOI:** 10.1186/s12917-016-0862-x

**Published:** 2016-10-10

**Authors:** Alana Christina Gast, Julia Metzger, Andrea Tipold, Ottmar Distl

**Affiliations:** 1 Institute for Animal Breeding and Genetics, University of Veterinary Medicine Hannover, Bünteweg 17p, 30559 Hannover, Germany; 2 Department of Small Animal Medicine and Surgery, University of Veterinary Medicine Hannover, Bünteweg 9, 30559 Hannover, Germany

**Keywords:** Hereditary ataxia, Association, *KCNJ10*, *CAPN1*, Parson Russell Terrier, Jack Russell Terrier

## Abstract

**Background:**

Spinocerebellar ataxia also referred to as hereditary ataxia comprises different forms of progressive neurodegenerative diseases. A complex mode of inheritance was most likely in Parson Russell Terriers (PRT) and in Jack Russell Terriers (JRT). Recently, the missense mutation *KCNJ10:*c.627C > G was shown to be associated with the spinocerebellar ataxia (SCA) in JRT and related Russell group of terriers, whereas the missense mutation *CAPN1:*c.344G > A was associated with late onset ataxia (LOA) in PRT.

**Results:**

We performed a genome-wide association study (GWAS) in PRT including 15 cases and 29 controls and found a statistically strong signal in the genomic region on dog chromosome 38 (CFA38) where *KCNJ10* is located. We tested the *CAPN1:*c.344G > A and *KCNJ10:*c.627C > G (Transcript XM_545752.4) mutations in a sample of 77 PRT and 9 JRT from Germany as well as further 179 controls from 20 different dog breeds. All cases and controls genotyped carried the wild-type for the *CAPN1:*c.344G > A mutation. Among the PRT, 17/77 (22.1 %) dogs were homozygous for the mutant *KCNJ10* allele and 22/77 (28.6 %) dogs were heterozygous. Three cases of PRT had the homozygous *KCNJ10* wild-type. In JRT, 1/3 cases did show the mutant *KCNJ10* allele homozygous. Thus, we sequenced the *KCNJ10* exons with their adjacent regions from 10 PRT and 3 JRT including the animals with imperfect co-segregation of the c.627C > G mutation. We identified a total of 45 genetic variants within *KCNJ10*. The most likely variant explaining the cases appeared a 1-bp-insertion in a C-stretch within exon 3 (*KCNJ10:*g.22141027insC). In silico analysis showed that this indel may influence the regulation of gene expression.

**Conclusions:**

In the present study, 16/21 cases of hereditary ataxia perfectly co-segregated with the *KCNJ10:*c.627C > G mutation. The *CAPN1:*c.344G > A mutation could not be validated and seems to be a rare variant in the samples screened. Screening *KCNJ10* for further mutations did result in a genetic variant explaining 2 JRT cases but further 3 cases with a non-mutant homozygous c.627C > G genotype could not be resolved. Breeders have to be aware that DNA-testing for hereditary ataxia in PRT and JRT does not capture all cases of hereditary ataxia in these dog breeds. At least one further form of hereditary ataxia not yet resolved by a mutation may occur in PRT and JRT.

**Electronic supplementary material:**

The online version of this article (doi:10.1186/s12917-016-0862-x) contains supplementary material, which is available to authorized users.

## Background

In dogs, spinocerebellar ataxia (hereditary ataxia) is characterized by progressive incoordination of gait, loss of balance, hypermetric and spastic movements. This condition may be associated with changes of brainstem auditory evoked potentials, myokymia, neuromyotonia and muscle fasciculation or seizures [1–7].

Hereditary ataxia is classified according to different aspects like age of onset (neonatal, juvenile and adult) and location of the pathomorphological lesions (spinal cord and/or cerebellar cortex) [8]. In Parson (PRT) and Jack Russell Terriers (JRT) lesions were found in the spinal cord and in the brain [2]. In Fox Terriers, lesions were only present in the spinal cord [9], whereas a more recent study reported histopathological degenerative changes in the brainstem [10]. Histopathological examinations of the central nervous system in PRT and JRT demonstrated a bilateral symmetrical myelopathy, predominantly a neuronal axonopathy in the dorsal and ventral or ventromedial funiculi [1, 2]. Loss of axons and myelin and astrogliosis were primarily seen in the spinocerebellar tracts of the cervical cord but were also present in all parts of the brain [1, 2, 7].

A complex segregation analysis using regressive logistic models was performed to elucidate the mode of inheritance in PRT and JRT [2]. The data included 3 pedigrees with a total of 115 dogs, either JRT or PRT, and 27 affected with the typical clinical signs and 9/27 with a histopathological examination showing a bilateral symmetric myelopathy in the nervous system. A monogenic mode of inheritance could be clearly ruled out. Mixed models with a major gene effect and a polygenic model were most likely for these pedigrees. The analysis could not differentiate how many loci may segregate in these two dog breeds.

In JRT, PRT and related breeds, the missense mutation c.627C > G in the *KCNJ10* (*potassium inwardly-rectifying channel, subfamily J*, *member 10)* gene (Transcript XM_545752.4) was found to be strongly associated with spinocerebellar ataxia (SCA) [7, 11]. This *KCNJ10* variant was identified screening a whole-genome sequence of a single JRT with spinocerebellar ataxia and myokymia under the assumptions of an autosomal recessive inheritance and a mutation altering the protein structure. Data filtering included other 81 canids that had not shown SCA. Affected animals showed ataxia, myokymia and/or seizures beginning from 2–12 months of age. The spinal cord sections revealed a bilateral myelopathy. Brain magnetic resonance imaging showed no abnormalities and hearing loss was not evident [7].

A missense mutation in the *CAPN1* gene (c.344G > A) was associated with a late-onset spinocerebellar ataxia phenotype (LOA) in a cohort of PRT [12]. Signs of symmetric spinocerebellar ataxia had been noticed in affected dogs between 6 and 12 months of age. The *CAPN1* missense mutation c.344G > A was identified using a genome-wide association study (GWAS) followed by the massively parallel sequencing of affected and control PRT for the LOA-associated chromosomal interval.

The objectives of this study were to perform a genome-wide association study (GWAS) in PRT for hereditary ataxia using the canine Illumina high density bead chip and then to show whether the previously reported SCA- and LOA-associated *KCNJ10* and *CAPN1* genes in PRT and JRT are in windows of significantly associated genomic regions. In addition, we sequenced *KCNJ10* and validated the previously reported *KCNJ10* and *CAPN1* mutations in PRT and JRT and further control samples of other breeds of dog.

## Methods

We collected EDTA-blood samples from 77 PRT and 9 JRT whereof 16 PRT and 3 JRT were clinically affected by SCA (Additional file 1). All 19 cases were presented at the clinic for Small Animals, University of Veterinary Medicine Hannover. Controls did not show any signs of ataxia, myokymia or seizures based on medical records of practitioners and clinical examinations at the clinic for Small Animals, Hannover. In addition, controls had to be >4 years of age. For 17 PRT a pedigree could be constructed. A subsample of the cases for the present study had been previously described elsewhere [2]. In brief, all clinically affected dogs showed symmetric generalized ataxia with hypermetric and spastic movements in all 4 legs at 2–9 months of age. Generalized seizures were observed in some cases. In all affected dogs clinical signs worsened and had to be euthanized at an age <3.5 years. All dogs had their origin in Germany. Out of the 16 clinically SCA-affected PRT cases, 14 were confirmed through a histopathological examination. In all examined 14 PRT, a bilateral symmetrical myelopathy was evident. Predominantly an axonopathy combined with myelin loss, in the dorsal and ventral or ventromedial funiculi was seen. Swelling of axons and dilatation of myelin sheaths with loss of myelin adjacent to a mild astrogliosis were observed primarily in the spinocerebellar tracts of the cervical cord but were also observed in all parts of the brain.

Genomic DNA was isolated using standard methods with RBC (Red Blood Cell) lysis buffer and SE (sodium EDTA) buffer. The DNA concentration of the samples was adjusted to 50 ng/μl using the Nanodrop ND-1000 (Peqlab Biotechnology, Erlangen, Germany) and quality control was performed by gel electrophoresis using 1 % agarose gels (peqGold Universal Agarose, Peqlab Biotechnologie).

We performed a GWAS in 44 PRT using the canine Illumina high density beadchip (Illumina, San Diego, CA, USA) containing 173,662 single nucleotide polymorphisms (SNPs). The data set included 15 cases and 29 controls. Out of these 15 cases, 12 had a histopathological examination confirming the clinical diagnosis. Controls had to be unrelated with the cases at the parent level and >4 years of age. Among controls, 11 were male and 18 female. Cases included 7 males and 8 females. After quality control (genotyping rate per SNP and animal >0.90), filtering for minor allele frequency >0.05 and Hardy-Weinberg equilibrium (HWE) (*p* < 0.000001) using SAS/Genetics, version 9.4 (Statistical Analysis System, SAS-Institute, Cary, NE, USA), 128,863 SNPs were left for the analysis.

The GWAS was performed with a general linear model. The analysis was run using TASSEL, version 3.0.164 [13]. The GLM explained for sex effects and the SNP genotypes. In addition, we tested several extended models employing up to five principal components (PCAs) to show the robustness of the outcome of the GWAS. All these models yielded the same genome-wide associated SNPs as the GLM model. Thus, adding principal components for cryptic data structure did not change the results of the GLM model. A plot of the first two PCAs for cases and controls employed for the GWAS is shown in Additional file 2. We applied the Bonferroni correction to determine the threshold for genome-wide significance in order to avoid false-positive associations. The Bonferroni correction set the threshold for significance at 0.05/128,863 = 3.88e^−7^ for a P-value of 0.05. A sex-stratified case-control analysis was performed using SAS/Genetics, version 9.4 (SAS). Homozygosity mapping was done in sliding windows with a minimum of 4 consecutive homozygous SNPs within a range of 50 kb using PLINK, version 1.07 [14].

A polymerase chain reaction-restriction fragment length polymorphism (PCR-RFLP) for a sample of 77 PRT, 9 JRT from Germany and 179 controls from 20 different dog breeds was employed for genotyping the *CAPN1:*c.344G > A and *KCNJ10:*c.627C > G mutations. The design of primers was carried out using Primer 3 (http://bioinfo.ut.ee/primer3-0.4.0/) and the dog genome assembly CanFam3.1 (Additional file 3).

The gene model we used for *KCNJ10* was based on the transcript XM_005640901.1 of the dog genome assembly CanFam3.1 (Additional file 4). This transcript includes three exons whereof only two exons (exon 2 and 3) are translated into a protein with 414 amino acids (aa). A previous study [7] used the transcript XM_545752.4 as gene model (denoted XM_545752.3 in previous reports [7, 11]) with two exons encoding 379 aa. Exon 2 and 3 of *KCNJ10* and their adjacent genomic regions were sequenced in 10 PRT (cases PRT I-III and PRT IV-VIII and controls PRT IX and PRT X) and 3 JRT (cases JRT I-III) using an ABI 3500 Genetic Analyzer (Applied Biosystems by Life Technologies, Darmstadt, Germany) (Additional file 3). In total, 45 genetic variants were detected in these 13 dogs. Exon 1 could not be amplified because several prime pairs designed for this canine genomic region or primer pairs designed from other species including human, mouse and rat did not result in amplicons with the expected size nor the amplicons could be sequenced. The exon 1 region did not seem to be correctly annotated in the dog reference genome and thus, may prevent retrieval of possible genetic variants for *KCNJ10* exon 1 in whole genome sequence data.

In addition, we tried to amplify the gene *KCNJ9* (gene ID 100855823) annotated at 22,100,627–22,104,165 base pairs (bp) on dog chromosome (CFA) 38 (CanFam3.1). The record of this gene contained large gaps. We searched for canine expressed sequence tags (ESTs) using the canine coding sequence of *KCNJ9* and the basic local alignment tool (blast) of NCBI (https://blast.ncbi.nlm.nih.gov/Blast.cgi). The best hit with an E-value of 1e^−77^ and a total score of 293 (DN374672.1) of this search did not fit to the genomic region where *KCNJ9* was annotated in the dog genome reference assembly. The same was found for hits with larger E-values. Therefore, we were not able to build a gene model for *KCNJ9* or sequence this gene.

All *KCNJ10* genetic variants detected in the 10 PRT and 3 JRT were further evaluated by sequencing *KCNJ10* from one dog of 9 other breeds. These dogs had a normal phenotype in regard to SCA and included the following breeds: Akita, Bernese mountain dog, Dalmatian, German Drahthaar, German shepherd, Irish wolfhound, Shar Pei, Tibetan terrier and Briard. Analysis of sequencing results had been performed using Sequencher software, version 4.8 (Gene Codes, Ann Arbor, MI, USA). The evaluation study resulted in 16 private variants for PRT and JRT. We then filtered the 16 genetic variants for homozygous mutant genotypes in affected PRT and JRT. The single nucleotide variant (SNV) reported in a previous study [7] and only one other variant (*KCNJ10:*g.22141027insC) remained. Validation of this result was performed in further 17 PRT. We developed a test on a LI-COR 4300 DNA Analyzer (LI-COR Biosciences, Lincoln, NE, USA) using a 167-bp amplicon (Additional file 3). The forward primer was labelled with IRD700 at the 5′end. All samples of the 77 PRT and 9 JRT as well as 84 dogs from 40 other different breeds were used to generate the *KCNJ10:*g.22141027insC containing amplicon. The amplicons were size-fractionated using gel electrophoresis on an automated sequencer LI-COR 4300 DNA Analyzer on 6 % polyacrylamide denaturing gels. Allele sizes were scored against IRD700-labeled DNA ladders (Additional file 5). For bioinformatic analysis of the effects of the *KCNJ10:*g.22141027insC variant we used the complete fasta-sequence of *KCNJ10* and employed the Regulatory RNA Motifs and Elements Finder (RegRNA) (http://regrna.mbc.nctu.edu.tw/html/about.html) [15] and the variant effect predictor (http://www.ensembl.org/Homo_sapiens/Tools/VEP?db=core).

## Results

### Genome-wide association study

The GWAS using 15 cases and 29 controls identified three genome-wide significantly associated SNPs on dog chromosome 38 (CFA38) (Additional file 6) and a quantile-quantile plot (Q-Q plot) for expected versus observed –log_10_ *P*-values was calculated to control for population stratification (Additional file 7). All SNPs with –log_10_P-values >5 are located on CFA38 and were in HWE. The genomic inflation factor was equal to 1, indicative that data stratification had no effect on the outcomes of the GWAS. The significantly associated SNPs are located at 18.53, 25.21 and 25.31 Mb (CanFam2) and at 15.53, 22.19 and 22.30 Mb (CanFam3.1) on CFA38 with the strongest signal at 22.30 Mb (P_raw_ = 5.14×10^−9^). The *P*-values for the SNPs with the highest associations after Bonferroni-correction were at 0.031 (BICF2G63072533), 0.036 (BICF2P378938) and 6.6×10^−4^ (BICF2P478530) (Additional file 8). In the associated interval, 9/15 cases and one control shared a homozygous region over 147 consecutive SNPs spanning 1893 kb at 23,387,952–25,280,713 bp (CanFam2) and at 20,388,358–22,267,778 (CanFam3.1). Inclusion of further two cases resulted in an even shorter homozygous interval with a size of 124 kb. However, for all other cases, an extended homozygosity region could not be determined (Additional file 9). This may indicate that hereditary ataxia had its origin in several founders or recombinations broke down the homozygous identical-by-descent region caused by a common founder to a very small region not detectable via the beadchip data.

A potential candidate gene in the associated and homozygous interval is *KCNJ10* at 25,148,668–25,149,816 bp (CanFam2) and at 22,114,718–22,143,618 bp (CanFam3.1) [7, 11]. The two closest and significantly associated SNPs are 58,212 (BICF2P378938) and 149,816 (BICF2P478530) bp downstream to *KCNJ10*. An association was not evident for SNPs on CFA18 where *CAPN1* is located. The gene *KCNJ9* could not be verified using expressed sequence tags (ESTs) for searching this region. Other candidate genes could not be detected in the associated interval. Further members of the *KCNJ*-gene family were not considered because the GWAS did not indicate significant signals on locations outside CFA38.

### Genotyping *CAPN1* and *KCNJ10* variants

*CAPN1* genotyping results showed that all 77 PRT and 9 JRT were homozygous wild-type. Genotyping the *KCNJ10:*c.627C > G mutation revealed 38/77 PRT (49.4 %) with the wild-type, 22/77 (28.6 %) as heterozygous and 17/77 (22.1 %) as homozygous mutated. In 1/38 of the homozygous wild-type tested PRT, hereditary ataxia was clinically and histopathologically confirmed. Two other homozygous wild-type tested PRT were diagnosed to be affected after clinical examination and/or according to the report of the owner. Among JRT, only 1/9 dogs was homozygously affected and 7/9 heterozygous. Each one of the heterozygous and homozygous wild-type JRT were clinically affected (Table 1). In addition, 179 controls from 20 different dog breeds were genotyped for both mutations. All these dogs were homozygous wild-type for both mutations (Additional file 10).

**Table 1:** Distribution of ataxia phenotypes by the *KCNJ10:*c.627C > G genotypes for Parson Russell (PRT) und Jack Russell Terriers (JRT)

Breed	Phenotype		*KCNJ10:*c.627C > G		
		*n*	C/C	C/G	G/G
PRT	A	12	1	0	11
	B	4	1	0	3
	C	2	1	0	1
	D	20	7	13	0
	E	3	1	2	0
	F	36	27	7	2
Subtotal		77	38	22	17
JRT	A	0	0	0	0
	B	3	1	1	1
	C	0	0	0	0
	D	0	0	0	0
	E	0	0	0	0
	F	6	0	6	0
Subtotal		9	1	7	1

A: hereditary ataxia clinically and histopathologically diagnosed, B: clinical signs of ataxia, C: clinical signs of ataxia, according to the medical records of the veterinary clinic and the report of the owner, D: unaffected dogs, E: unaffected dogs, but affected dogs in progeny or siblings, F: no information on health status; the phenotype group A contains 4 PRT shown in the pedigree (Pedigree numbers (P.n.) 14, 15, 16, 17, see Additional file 12), group D contains 7 PRT (P.n. 2, 6, 7, 8, 9, 10, 11, see Additional file 12) and group F includes 6 PRT (P.n. 1, 3, 4, 5, 12, 13, see Additional file 12)

### Sequencing *KCNJ10*

In order to identify further mutations associated with hereditary ataxia explaining the cases with the homozygous *KCNJ10* C/C genotype, exon 2 and 3 of *KCNJ10* with their exon/intron or exon/UTR boundaries were sequenced in PRT and JRT. We included 3 affected PRT with the homozygous *KCNJ10* C/C genotype and all 3 affected JRT with the different *KCNJ10* genotypes. In addition, we sequenced 7 PRT (5 affected PRT and 2 unaffected) with the concordant *KCNJ10* genotype. A total of 45 genetic variants could be identified including 41 single nucleotide variants (SNVs) and 4 indels (3 insertions and 1 deletion) (Additional file 11) whereof most of them were located in the 3′UTR (CanFam3.1) (Additional file 4). Sequencing further 9 dogs of other breeds (Additional file 12) and filtering showed 16 variants private for PRT and JRT (Additional file 13). A further filtering for homozygous mutant genotypes in affected PRT and JRT resulted in a 1-bp-insertion (*KCNJ10:*g.22141027insC) prevalent in these animals. This 1-bp-insertion was located within a stretch of 7 consecutive C bases (Additional file 5). Bioinformatic analysis using RegRNA indicated the *KCNJ10:*g.22141027insC affecting regulation of gene expression via a regulatory RNA motif or miRNA target site. A regulatory RNA motif (5′-GGGAGGGG-3′) was identified 6 bp downstream to the *KCNJ10:*g.22141027insC. The motif of the miRNA (hsa-miR-1180) target site contains the *KCNJ10:*g.22141027insC (5′-acACAACCCCCCCGACGCCGGGAg-3′). Genotyping a pedigree containing 17 PRT for both mutations, *KCNJ10:*g.22141027insC and *KCNJ10:*c.627C > G, showed segregation of both mutations with the affection status with exception of one animal (Additional file 14). Genotyping results of the *KCNJ10:*g.22141027insC are summarized in Table 2. Out of the 77 PRT, 34 (44.2 %) showed the wild-type, 26 (33.8 %) the heterozygous and 17 (22.1 %) the homozygous mutated genotype. In 2/26 of the heterozygous PRT, hereditary ataxia was clinically and histopathologically confirmed and 2/34 homozygous wild-type tested PRT were affected according to a clinical examination and/or according to the report of the owner. The genotyping results for the *KCNJ10:*g.22141027insC mutation of JRT showed two homozygous mutated genotypes among the affected individuals. A further affected individual had the wildtype genotype. Genotyping further 84 dogs of 40 other breed dogs revealed 59 (70.3 %) wildtype homozygous, 21 (25.0 %) heterozygous and 4 (4.8 %) homozygous mutated genotypes (Additional file 15).

**Table 2:** Distribution of ataxia phenotypes by the *KCNJ10:g.22141027insC* genotypes for Parson Russell (PRT) und Jack Russell Terriers (JRT)

Breed	Phenotype		*KCNJ10:*g.22141027insC		
		*n*	wt/wt	wt/mut	mut/mut
PRT	A	12	0	2	10
	B	4	1	0	3
	C	2	1	0	1
	D	20	7	12	1
	E	3	1	2	0
	F	36	24	10	2
Subtotal		77	34	26	17
JRT	A	0	0	0	0
	B	3	1	0	2
	C	0	0	0	0
	D	0	0	0	0
	E	0	0	0	0
	F	6	0	6	0
Subtotal		9	1	6	12

A: hereditary ataxia clinically and histopathologically diagnosed, B: clinical signs of ataxia, C: clinical signs of ataxia according to the medical records of the veterinary clinic and report of the owner, D: unaffected dogs, E: unaffected dogs, but affected dogs in progeny or siblings, F: no information on health status; the phenotype group A contains 4 PRT shown in the pedigree (Pedigree numbers (P.n.) 14, 15, 16, 17, see Additional file 12), group D contains 7 PRT (P.n. 2, 6, 7, 8, 9, 10, 11, see Additional file 12) and group F includes 6 PRT (P.n. 1, 3, 4, 5, 12, 13, see Additional file 12)

## Discussion

The results of the GWAS indicate that the associated genomic region on CFA38 may harbour the most likely candidate genes for hereditary ataxia in the sample under study. Thus, our study corroborated the previous candidate gene search using whole genome sequence data [7]. We could not detect samples with the *CAPN1:*c.344G > A mutation. This may indicate that this mutation may be rare in the German PRT and JRT populations. In agreement with a previous study [7], one histopathologically confirmed case of hereditary ataxia and two clinically affected cases carried neither the *CAPN1:*c.344G > A allele nor the homozygous *KCNJ10:*c.627C > G genotype. This may indicate that at least one further mutation may be involved in hereditary ataxia of PRT and JRT. As the largest proportion of affected dogs were concordant with the segregation of the *KCNJ10:*c.627C > G alleles the remaining unexplained cases may harbour a rare mutation. However, breeders have to be aware that selective breeding based on genetic tests for the *KCNJ10:*c.627C > G and *CAPN1:*c.344G > A mutations cannot completely preclude affected dogs of the breeds PRT or JRT despite of homozygous wild-type genotypes of the parents.

The present sequence analysis tagged a 1-bp-insertion associated with hereditary ataxia in PRT and JRT. This mutation may have an effect on regulation of *KCNJ10* expression as indicated through bioinformatic results from RegRNA. However, the presence of this 1-bp-insertion in other breeds may require further research in phenotyping spinocerebellar ataxia associated with myokymia, seizures, or both. In conclusion, a few but not all cases of hereditary ataxia being heterozygous or homozygous for the wild-type allele of *KCNJ10* could be explained by the *KCNJ10:*g.22141027insC mutation.

## Conclusions

Our data confirmed the presence of the *KCNJ10:*c.627C > G mutation in PRT and JRT affected with hereditary ataxia. However, some cases of hereditary ataxia could not be explained through the *KCNJ10:*c.627C > G nor the *CAPN1:*c.344G > A mutation. Genetic testing using the *KCNJ10:*c.627C > G and the *CAPN1:*c.344G > A mutations deems not to be sufficient to capture all cases of hereditary ataxia in PRT and JRT. This lets us assume that at least one additional form of hereditary ataxia may segregate in the breeds PRT and JRT. The *KCNJ10:*g.22141027insC mutation was evident in a few cases but could not explain all not yet resolved cases. Further efforts are necessary to collect cases of hereditary ataxia not explained by the presently known mutations to unravel the responsible mutations in these cases of hereditary ataxia.

## Additional files

Additional file 1: Distribution of ataxia phenotypes for Parson Russell (PRT) and Jack Russell Terriers (JRT). (DOCX 15 kb)
Additional file 2: Plot of the first (PCA1) versus the second principal component (PCA2) by hereditary ataxia affected Parson Russell Terriers (affected) and controls (non-affected) used for the genome-wide association study (GWAS). The black dots indicate controls and the orange dots affected PRT. Number of single nucleotide polymorphisms (SNPs) that informed PCAs was at 128,863 SNPs. Variance of SNPs explained by PCA1 and PCA2 was at 0.19 and 0.17, respectively. (DOC 69 kb)
Additional file 3: Primer pair sequences, restriction enzymes, annealing temperature (AT) and amplicon size used for genotyping the mutations *CAPN1:*c.344G > A and *KCNJ10:*c.627C > G by PCR-RFLP are given. Primer pair sequences, annealing temperature (AT) and amplicon size of PCR for genotyping the mutation *KCNJ10:*g.22141027insC validated on a LI-COR 4300 DNA Analyzer as well as primer pair sequences and product-sizes for Sanger sequencing PCR-amplicons of exon 2 and 3 of *KCNJ10* are shown. (DOC 57 kb)
Additional file 4: Canine gene model of *KCNJ10* and detected variants. The gene model was built using the Ensemble annotation (assembly CanFam3.1). (A) represents transcript XM_005640901.1 and (B) represents XM_545752.4. Translated exons are shown as black boxes and untranslated exons are shown as open boxes. Exon numbers are given above the boxes. Continuous lines indicate introns. Sizes of exons and introns are given below the boxes and lines. Positions of start codon and stop codon are also present. Locations and motifs of variants identified in our study are listed above the exons. Both the single nucleotide variant (SNV) reported by Gilliam et al. [7] (*KCNJ10:*c.627C > G) and the variant validated in our study (*KCNJ10:*g.22141027insC) are framed by a black open box. (PDF 41 kb)
Additional file 5: (A) The genomic sequences of an unaffected and affected Parson Russell Terrier (PRT) are shown. The unaffected dog is homozygous for the wild-type allele. The sequence of the dog affected by hereditary ataxia shows a 1-base pair (bp) insertion in a seven-C stretch within exon 3 of the *KCNJ10* gene*.* The seven-C stretch with and without the inserted base C is framed by a red open box*.* The homozygous wild-type and the homozygous mutant variant in the forward sequence as well as the heterozygous variant in both the forward and reverse sequence are shown. (B) Fragment length analysis on a 6 % polyacrylamide gel for evaluating the 1-bp insertion in *KCNJ10* in hereditary ataxia affected Parson and Jack Russell Terriers. The homozygous wild-type (wt/wt) in two unaffected PRT are shown. The size of the normal PCR-product is 166 bp. The heterozygous genotype (wt/mut) in one unaffected and the homozygous genotype (mut/mut) for the mutant allele in two affected PRT are also present. (PDF 169 kb)
Additional file 6: Manhattan plot of –log_10_ *P*-values of the genome-wide association study for hereditary ataxia in Parson Russell Terriers using a general model analysis. On the X-axis, the SNPs are given by dog chromosome number. The –log_10_P-values for each SNP genotype effect are plotted against the SNP position on each chromosome. Chromosomes are differentiated by colors. The color keys are given below the plot. The blue line indicates the threshold of the –log_10_ *P*-values for genome-wide significance after Bonferroni correction. (PDF 138 kb)
Additional file 7: Q-Q-plot of expected –log_10_ *P*-values versus observed–log_10_ *P*-values from the general model analysis for hereditary ataxia in Parson Russell Terriers. Shown are all 128,863 SNPs included in the genome-wide association analysis with the grey line corresponding to the null hypothesis of no association. (PDF 56 kb)
Additional file 8: Summary of results for the genome-wide association study using a general model analysis and sex-stratified case-control analysis for hereditary ataxia in Parson Russell Terriers. The SNP-ID, the position on dog chromosome (CFA) in base pairs (bp) according to the dog genome assembly CanFam2, minor allele, minor allele frequency (MAF) for all, affected (MAF_A_) and unaffected (MAF_U_) dogs (controls), variance explained by the single SNP (V_SNP_), −log_10_ *P*-values (−log_10_P) and Bonferroni-corrected -log_10_ *P*-values (−log_10_P_Bonf_) of the general model analysis are given. Odds ratios (OR) with 95 % confidence intervals (CI) are from a sex-stratified case-control analysis for cases and controls. (DOC 42 kb)
Additional file 9: Overview on the results of extended homozygosity mapping in Parson Russell Terriers (PRT) genotyped on the canine Illumina high density beadchip. The IDs of PRT, *KCNJ10* c.627C > G genotype, the SNP-IDs and positions in base pairs (bp) for the start and end of the homozygosity region are given. The PRT-ID 2 indicates dogs which were used for sequencing in this study and PRT-ID 3 indicates the designation for PRT (P.n.) which are shown in the pedigree (see Additional file 12). *KCNJ10* is located at 25,148,668–25,149,816 on CFA38 (CanFam2). (DOC 51 kb)
Additional file 10: Distribution of genotyping results for the mutation *CAPN1:*c.344G > A and *KCNJ10:*c.627C > G in different dog breeds. Numbers of tested homozygous wild-type (wt/wt), heterozygous (wt/mut) and homozygous mutant (mut/mut) genotypes are given. (DOC 51 kb)
Additional file 11: Identification of variants in the gene *KCNJ10* in affected and unaffected Parson Russell Terriers (PRT) and Jack Russell Terriers (JRT). IDs of the variants, locations in the gene, accession numbers and the genotype for each variant are given. The single nucleotide variant reported by Gilliam et al. [7] (*KCNJ10:*c.627C > G) written in bold. Indel mutations are denoted as wt (wild-type) or mut (mutant). (DOC 108 kb)
Additional file 12: Sequencing results of nine dogs of different breeds for evaluation of the variants detected in Parson Russell Terriers (PRT) and Jack Russell Terriers (JRT). IDs of the variants and the genotype for each variant are given. The single nucleotide variant reported by Gilliam et al. [7] (*KCNJ10:*c.627C > G) written in bold. Indel mutations are denoted as wt (wildtype) or mut (mutant). Indel mutations are denoted as wt (wild-type) or mut (mutant). (DOC 97 kb)
Additional file 13: Variants in the gene *KCNJ10* which are private for Parson Russell Terriers (PRT) and Jack Russell Terriers (JRT). IDs of the variants, locations in the gene, accession numbers and the genotype for each variant are given. Both the single nucleotide variant reported by Gilliam et al. [7] (*KCNJ10:*c.627C > G) and *KCNJ10:*g.22141027insC identified as further putative variant written in bold. Indel mutations are denoted as wt (wild-type) or mut (mutant). (DOC 64 kb)
Additional file 14: The pedigree demonstrates the relationship of 17 Parson Russell Terriers (PRT) (pedigree number 1–17) selected from the sample of our study. Genotypes for both, the *KNCJ10:*g.22141027insC (first row) and *KCNJ10:*c.627C > G [7] (second row) variant are given for each of the 17 dogs. *KNCJ10:*g.22141027insC genotyping resulted in 5/17 wild-type (wt/wt), 9/17 heterozygous (wt/mut) and 3/17 homozygous mutant (mut/mut) PRT. *KCNJ10:*c.627C > G genotyping resulted in 9/17 wild-type (wt/wt), 5/17 heterozygous (wt/mut) and 3/17 homozygous mutant (mut/mut) PRT. (PDF 22 kb)
Additional file 15: Distribution of genotyping results for the mutation *KCNJ10:*g.22141027insC in different dog breeds. Numbers of tested homozygous wild-type (wt/wt), heterozygous (wt/mut) and homozygous mutant (mut/mut) genotypes are given. (DOC 57 kb)

## Abbreviations

CFA: Dog chromosome
GWAS: Genome-wide association study
JRT: Jack Russell Terrier
*KCNJ10*: *Potassium inwardly-rectifying channel, subfamily J*, *member 10*
LOA: Late onset ataxia
PRT: Parson Russell Terrier
SCA: Spinocerebellar ataxia
SNP: Single nucleotide polymorphism
SNV: Single nucleotide variant
