# Supplementary material for: Genome-wide association study for hereditary ataxia in the Parson Russell Terrier and DNA-testing for ataxia-associated mutations in the Parson and Jack Russell Terrier
Source: BMC Vet Res. 2016 Oct 10;12:225. doi: 10.1186/s12917-016-0862-x (PMC5057501; doi:10.1186/s12917-016-0862-x)
Supplement: Additional file 1: — Distribution of ataxia phenotypes for Parson Russell (PRT) and Jack Russell Terriers (JRT). (DOCX 15 kb) [file 12917_2016_862_MOESM1_ESM.docx]

**Additional file 1:** Distribution of ataxia phenotypes for Parson Russell (PRT) and Jack Russell Terriers (JRT)

| Breed | Phenotype | n | Male | Female | Number of dogs included in a pedigree |
| --- | --- | --- | --- | --- | --- |
| PRT | A | 12 | 8 | 4 | 4 |
|  | B | 4 | 2 | 2 |  |
|  | C | 2 | 2 | 0 |  |
|  | D | 20 | 9 | 11 | 7 |
|  | E | 3 | 0 | 3 |  |
|  | F | 36 | 20 | 16 | 6 |
| Subtotal |  | 77 | 41 | 36 | 17 |
| JRT | A | 0 | 0 | 0 |  |
|  | B | 3 | 2 | 1 |  |
|  | C | 0 | 0 | 0 |  |
|  | D | 0 | 0 | 0 |  |
|  | E | 0 | 0 | 0 |  |
|  | F | 6 | 1 | 5 |  |
| Subtotal |  | 9 | 3 | 6 |  |

A: hereditary ataxia clinically and histopathologically diagnosed, B: clinical signs of ataxia, C: clinical signs of ataxia, according to the medical records of the veterinary clinic and the report of the owner, D: unaffected dogs, E: unaffected dogs, but affected dogs in progeny or siblings, F: no information on health status; the phenotype group A contains 4 PRT shown in the pedigree (Pedigree numbers (P.n.) 14, 15, 16, 17, see Additional file 14), group D contains 7 PRT (P.n. 2, 6, 7, 8, 9, 10, 11, see Additional file 14) and group F includes 6 PRT (P.n. 1, 3, 4, 5, 12, 13, see Additional file 14).
