# Supplementary material for: Genome-wide association study for hereditary ataxia in the Parson Russell Terrier and DNA-testing for ataxia-associated mutations in the Parson and Jack Russell Terrier
Source: BMC Vet Res. 2016 Oct 10;12:225. doi: 10.1186/s12917-016-0862-x (PMC5057501; doi:10.1186/s12917-016-0862-x)

**Additional file 2:** Plot of the first (PCA1) versus the second principal component (PCA2) by hereditary ataxia affected Parson Russell Terriers (affected) and controls (non-affected) used for the genome-wide association study (GWAS). The black dots indicate controls and the orange dots affected PRT. Number of single nucleotide polymorphisms (SNPs) that informed PCAs was at 128,863 SNPs. Variance of SNPs explained by PCA1 and PCA2 was at 0.19 and 0.17, respectively.


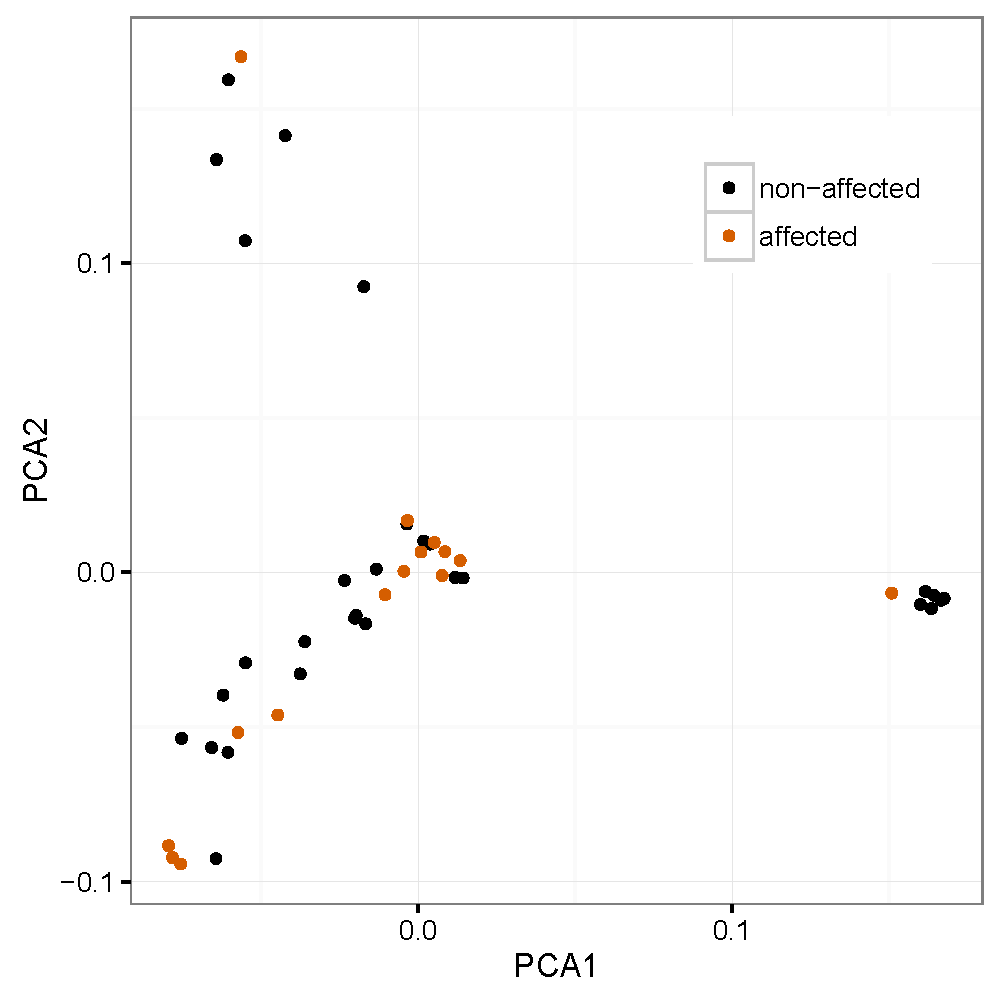

Supplement: Additional file 2: — Plot of the first (PCA1) versus the second principal component (PCA2) by hereditary ataxia affected Parson Russell Terriers (affected) and controls (non-affected) used for the genome-wide association study (GWAS). The black dots indicate controls and the orange dots affected PRT. Number of single nucleotide polymorphisms (SNPs) that informed PCAs was at 128,863 SNPs. Variance of SNPs explained by PCA1 and PCA2 was at 0.19 and 0.17, respectively. (DOC 69 kb) [file 12917_2016_862_MOESM2_ESM.doc]
