# Supplementary material for: Genome-wide association study for hereditary ataxia in the Parson Russell Terrier and DNA-testing for ataxia-associated mutations in the Parson and Jack Russell Terrier
Source: BMC Vet Res. 2016 Oct 10;12:225. doi: 10.1186/s12917-016-0862-x (PMC5057501; doi:10.1186/s12917-016-0862-x)
Supplement: Additional file 3: — Primer pair sequences, restriction enzymes, annealing temperature (AT) and amplicon size used for genotyping the mutations CAPN1:c.344G > A and KCNJ10:c.627C > G by PCR-RFLP are given. Primer pair sequences, annealing temperature (AT) and amplicon size of PCR for genotyping the mutation KCNJ10:g.22141027insC validated on a LI-COR 4300 DNA Analyzer as well as primer pair sequences and product-sizes for Sanger sequencing PCR-amplicons of exon 2 and 3 of KCNJ10 are shown. (DOC 57 kb) [file 12917_2016_862_MOESM3_ESM.doc]

**Additional file 3:** Primer pair sequences, restriction enzymes, annealing temperature (AT) and amplicon size used for genotyping the mutations *CAPN1:*c.344G>A and *KCNJ10:*c.627C>G by PCR-RFLP are given. Primer pair sequences, annealing temperature (AT) and amplicon size of PCR for genotyping the mutation *KCNJ10:*g.22141027insC validated on a LI-COR 4300 DNA Analyzer as well as primer pair sequences and product-sizes for Sanger sequencing PCR-amplicons of exon 2 and 3 of *KCNJ10* are shown.

| Mutation | Enzyme | Forward and reverse primer sequences | AT °C | Product-size |
| --- | --- | --- | --- | --- |
| *CAPN1:*c.344G>A | HpyCH4III | 5’CTGGTTTGCCTAGATTCCTG3’ | 59 | 259 bp |
|  |  | 5’AATGGAAGATGCCAGCATAG3’ |  |  |
| *KCNJ10:*c.627C>G | NlaIII | 5`GCCAACATGCGGAAGAGCCT3` | 60 | 233 bp |
|  |  | 5`TCGAAGTCACCCTCGCCACT3` |  |  |
| *KCNJ10:*g.22141027insC |  | 5`CTGGAGTCCCAGCTTTCCTTC3` | 61 | 167 bp |
|  |  | 5`TATCCATCCAACTGCACTGTC3` |  |  |
| PCR-Amplicon | Number of Amplicon |  |  |  |
| Exon 2 | 2 | 5`CGAGAAGATACGGGGATGAG3` | 60 | 566 bp |
|  |  | 5`GAGTAGATGGGCAGAAACAGG3` |  |  |
| Exon 3 | 3.1 | 5`TAAGTATTGGCCGACAGTGAG3` | 60 | 857 bp |
|  |  | 5`GTGTCGACCTGGAAAGTCAC3` |  |  |
| Exon 3 | 3.2 | 5`GACCCACCAGACCAAAGAG3` | 60 | 803 bp |
|  |  | 5`TATCCATCCAACTGCACTGTC3` |  |  |
| Exon 3 | 3.3 | 5`TGCTTGGAGACTCACATTAGG3` | 60 | 862 bp |
|  |  | 5`TAAGGGAGACCCTGGTTATTG3` |  |  |
| Exon 3 | 3.4 | 5`TGTGCTCCTCTGACAAGTGC3` | 60 | 593 bp |
|  |  | 5`GCTCTTTGCCTTCCATTCTG3` |  |  |
| Exon 3 | 3.5 | 5`AGCTCTCAAGGGAACAAGACC3` | 60 | 795 bp |
|  |  | 5`AGCCTCCCTATGTACCACGTC3` |  |  |
| Exon 3 | 3.6 | 5`GCAAAACAACAGATGGGAGAG3` | 60 | 853 bp |
|  |  | 5`GGTCCTGTTAGCTGGTGGAG3` |  |  |
