# Supplementary material for: Genome-wide association study for hereditary ataxia in the Parson Russell Terrier and DNA-testing for ataxia-associated mutations in the Parson and Jack Russell Terrier
Source: BMC Vet Res. 2016 Oct 10;12:225. doi: 10.1186/s12917-016-0862-x (PMC5057501; doi:10.1186/s12917-016-0862-x)

Additional file 4: Canine gene model of *KCNJ10* and detected variants. The gene model was built based using the Ensemble annotation (Assembly CanFam3.1). (A) represents transcript XM_005640901.1 and (B) represents XM_545752.4. Translated exons are shown as black boxes and untranslated exons are shown as open boxes. Exon numbers are given above the boxes. Continuous lines indicate introns. Sizes of exons and introns are given below the boxes and lines. Positions of start codon and stop codon are also present. Locations and motifs of variants identified in our study are listed above the exons. Both the single nucleotide variant (SNV) reported by Gilliam et al. (2014) (*KCNJ10:*c.627C>G) [7] and the variant validated in our study (*KCNJ10:*g.22141027insC) are framed by a black open box.

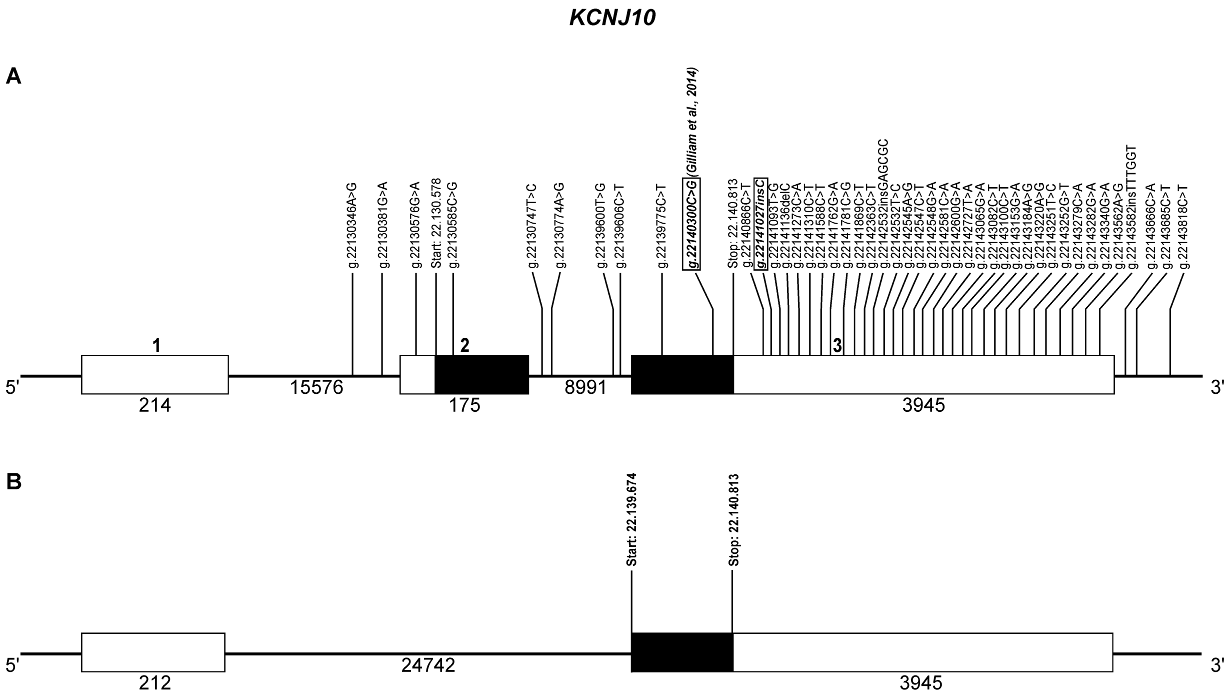

Supplement: Additional file 4: — Canine gene model of KCNJ10 and detected variants. The gene model was built using the Ensemble annotation (assembly CanFam3.1). (A) represents transcript XM_005640901.1 and (B) represents XM_545752.4. Translated exons are shown as black boxes and untranslated exons are shown as open boxes. Exon numbers are given above the boxes. Continuous lines indicate introns. Sizes of exons and introns are given below the boxes and lines. Positions of start codon and stop codon are also present. Locations and motifs of variants identified in our study are listed above the exons. Both the single nucleotide variant (SNV) reported by Gilliam et al. [7] (KCNJ10:c.627C > G) and the variant validated in our study (KCNJ10:g.22141027insC) are framed by a black open box. (PDF 41 kb) [file 12917_2016_862_MOESM4_ESM.pdf]
