# Supplementary material for: Genome-wide association study for hereditary ataxia in the Parson Russell Terrier and DNA-testing for ataxia-associated mutations in the Parson and Jack Russell Terrier
Source: BMC Vet Res. 2016 Oct 10;12:225. doi: 10.1186/s12917-016-0862-x (PMC5057501; doi:10.1186/s12917-016-0862-x)

Additional file 5: (A) The genomic sequences of an unaffected and an affected Parson Russell Terrier (PRT) are shown. The unaffected dog is homozygous for the wild-type allele. The sequence of the dog affected by hereditary ataxia shows a 1-base pair (bp) insertion in a seven-C stretch within exon 3 of the *KCNJ10* gene. The seven-C stretch with and without the inserted base C is framed by a red open box. The homozygous wild-type and the homozygous mutant variant in the forward sequence as well as the heterozygous variant in both the forward and reverse sequence are shown. (B) Fragment length analysis on a 6% polyacrylamide gel for evaluating the 1-bp insertion in *KCNJ10* in hereditary ataxia affected Parson and Jack Russell Terriers. The homozygous wild-type (wt/wt) in two unaffected PRT are shown. The size of the normal PCR-product is 166 bp. The heterozygous genotype (wt/mut) in one unaffected and the homozygous genotype (mut/mut) for the mutant allele in two affected PRT are also present.

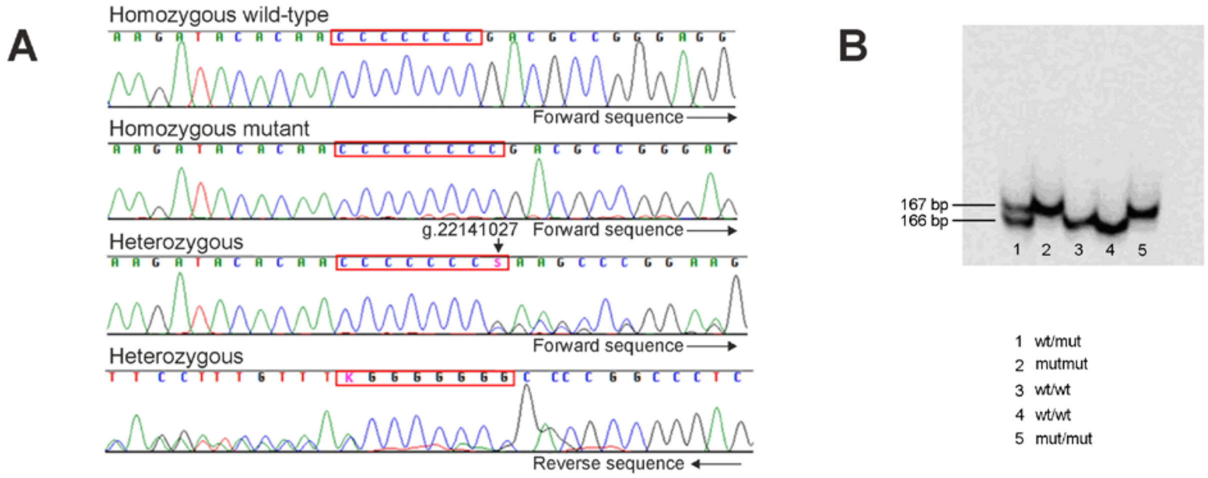

Supplement: Additional file 5: — (A) The genomic sequences of an unaffected and affected Parson Russell Terrier (PRT) are shown. The unaffected dog is homozygous for the wild-type allele. The sequence of the dog affected by hereditary ataxia shows a 1-base pair (bp) insertion in a seven-C stretch within exon 3 of the KCNJ10 gene. The seven-C stretch with and without the inserted base C is framed by a red open box. The homozygous wild-type and the homozygous mutant variant in the forward sequence as well as the heterozygous variant in both the forward and reverse sequence are shown. (B) Fragment length analysis on a 6 % polyacrylamide gel for evaluating the 1-bp insertion in KCNJ10 in hereditary ataxia affected Parson and Jack Russell Terriers. The homozygous wild-type (wt/wt) in two unaffected PRT are shown. The size of the normal PCR-product is 166 bp. The heterozygous genotype (wt/mut) in one unaffected and the homozygous genotype (mut/mut) for the mutant allele in two affected PRT are also present. (PDF 169 kb) [file 12917_2016_862_MOESM5_ESM.pdf]
