# Supplementary material for: Genome-wide association study for hereditary ataxia in the Parson Russell Terrier and DNA-testing for ataxia-associated mutations in the Parson and Jack Russell Terrier
Source: BMC Vet Res. 2016 Oct 10;12:225. doi: 10.1186/s12917-016-0862-x (PMC5057501; doi:10.1186/s12917-016-0862-x)

Additional file 6: Manhattan plot of $-\log_{10}$P-values of the genome-wide association study for hereditary ataxia in Parson Russell Terriers using a general model analysis. On the X-axis, the SNPs are given by dog chromosome number. The $-\log_{10}$P-values for each SNP genotype effect are plotted against the SNP position on each chromosome. Chromosomes are differentiated by colors. The color keys are given below the plot. The blue line indicates the threshold of the $-\log_{10}$P-values for genome-wide significance after Bonferroni correction.

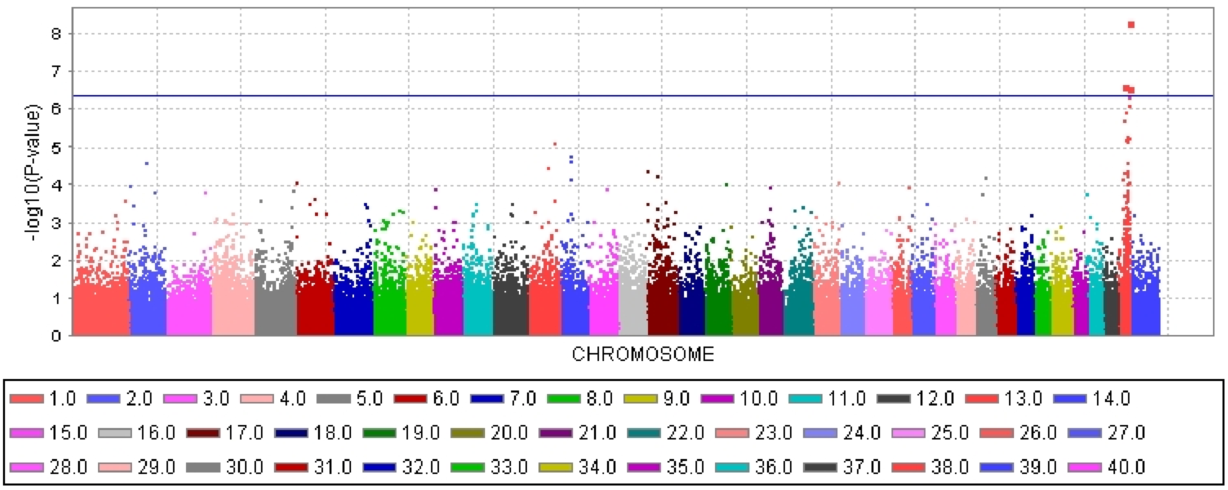

Supplement: Additional file 6: — Manhattan plot of –log10 P-values of the genome-wide association study for hereditary ataxia in Parson Russell Terriers using a general model analysis. On the X-axis, the SNPs are given by dog chromosome number. The –log10P-values for each SNP genotype effect are plotted against the SNP position on each chromosome. Chromosomes are differentiated by colors. The color keys are given below the plot. The blue line indicates the threshold of the –log10 P-values for genome-wide significance after Bonferroni correction. (PDF 138 kb) [file 12917_2016_862_MOESM6_ESM.pdf]
