# Supplementary material for: Genome-wide association study for hereditary ataxia in the Parson Russell Terrier and DNA-testing for ataxia-associated mutations in the Parson and Jack Russell Terrier
Source: BMC Vet Res. 2016 Oct 10;12:225. doi: 10.1186/s12917-016-0862-x (PMC5057501; doi:10.1186/s12917-016-0862-x)

Additional file 7: Q-Q-plot of expected –$\log_{10}$P-values versus observed–$\log_{10}$P-values from the general model analysis for hereditary ataxia in Parson Russell Terriers**.** (A) Shown are all 128,863 SNPs included in the genome-wide association analysis with the grey line corresponding to the null hypothesis of no association. (B) Shown are those SNPs from the genome-wide association analysis with a -$\log_{10}$-P-value < 5.

(A)

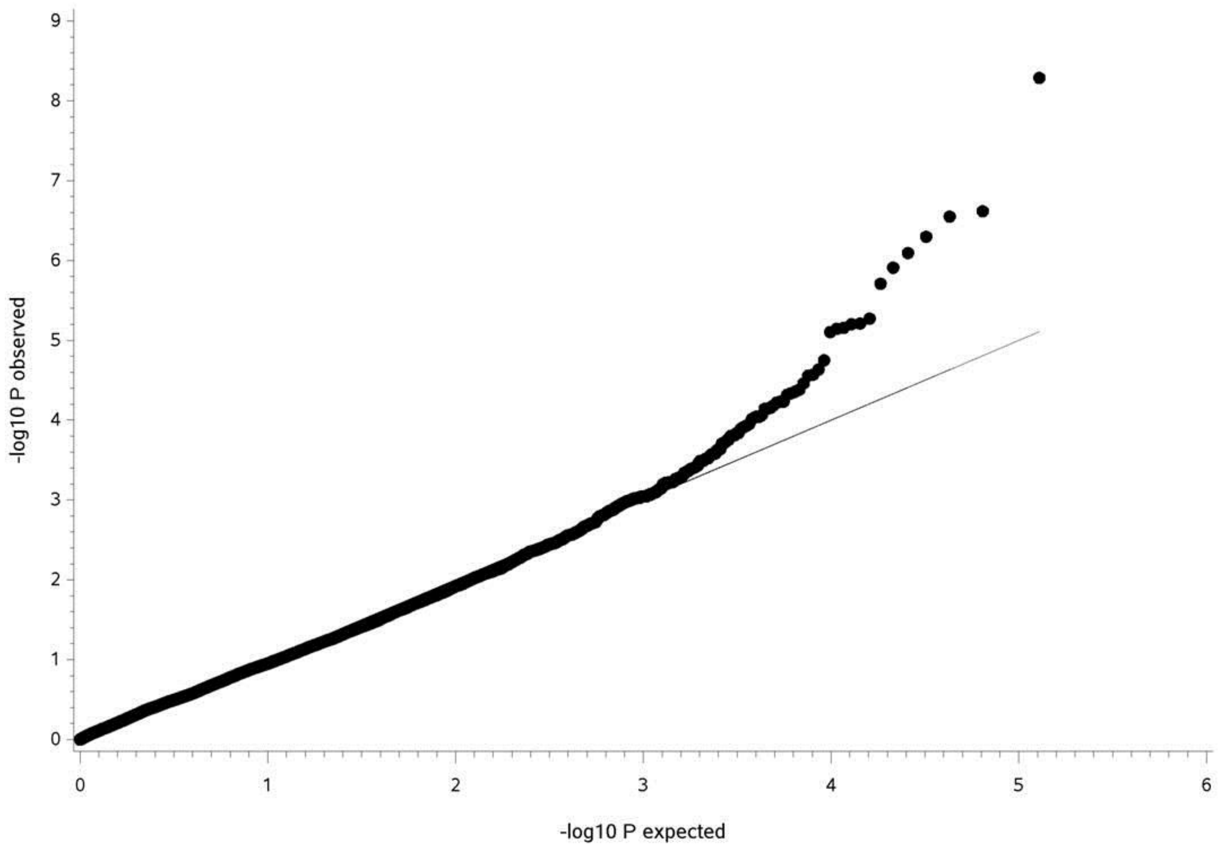

(B)

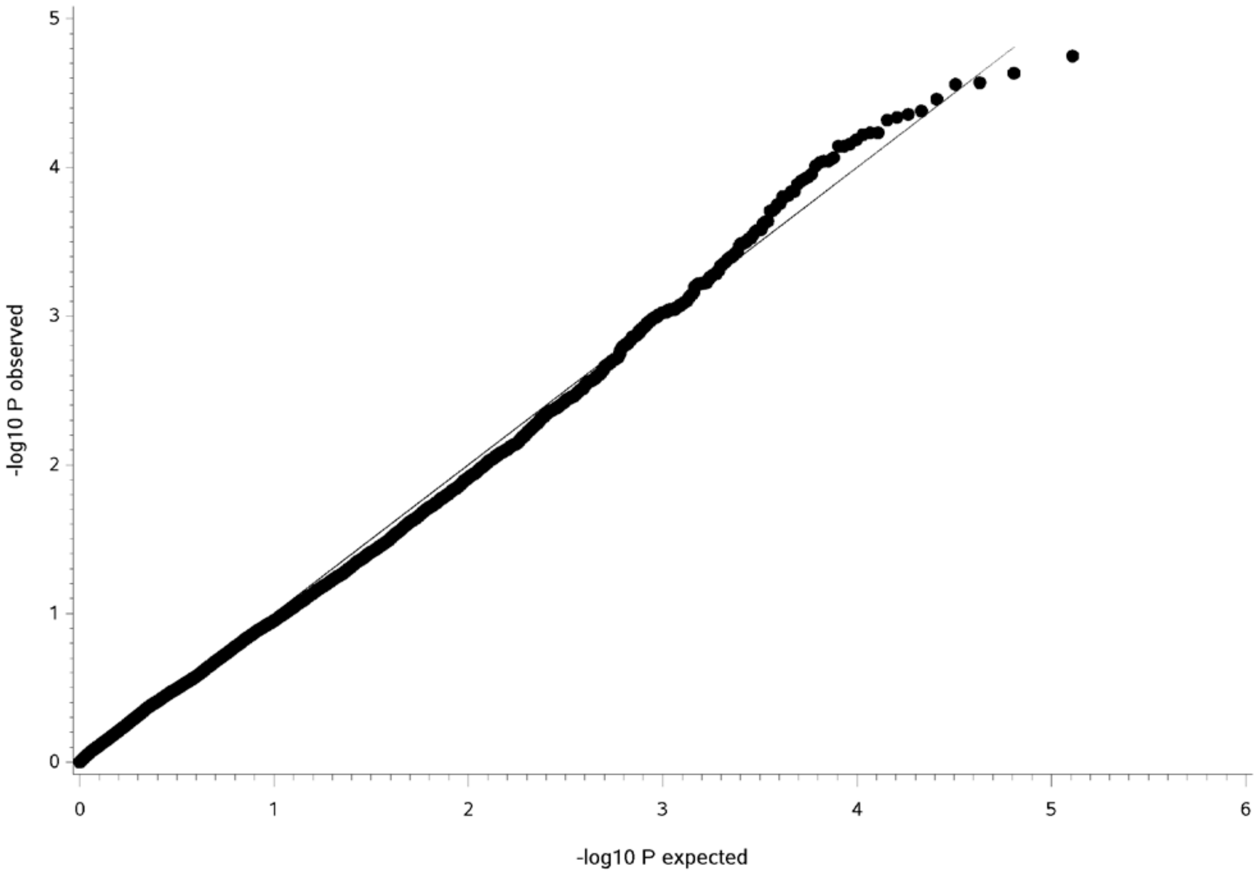

Supplement: Additional file 7: — Q-Q-plot of expected –log10 P-values versus observed–log10 P-values from the general model analysis for hereditary ataxia in Parson Russell Terriers. Shown are all 128,863 SNPs included in the genome-wide association analysis with the grey line corresponding to the null hypothesis of no association. (PDF 56 kb) [file 12917_2016_862_MOESM7_ESM.pdf]
