# Supplementary material for: Genome-wide association study for hereditary ataxia in the Parson Russell Terrier and DNA-testing for ataxia-associated mutations in the Parson and Jack Russell Terrier
Source: BMC Vet Res. 2016 Oct 10;12:225. doi: 10.1186/s12917-016-0862-x (PMC5057501; doi:10.1186/s12917-016-0862-x)
Supplement: Additional file 8: — Summary of results for the genome-wide association study using a general model analysis and sex-stratified case-control analysis for hereditary ataxia in Parson Russell Terriers. The SNP-ID, the position on dog chromosome (CFA) in base pairs (bp) according to the dog genome assembly CanFam2, minor allele, minor allele frequency (MAF) for all, affected (MAFA) and unaffected (MAFU) dogs (controls), variance explained by the single SNP (VSNP), −log10 P-values (−log10P) and Bonferroni-corrected -log10 P-values (−log10PBonf) of the general model analysis are given. Odds ratios (OR) with 95 % confidence intervals (CI) are from a sex-stratified case-control analysis for cases and controls. (DOC 42 kb) [file 12917_2016_862_MOESM8_ESM.doc]

**Additional file 8:** Summary of results for the genome-wide association study using a general model analysis and sex-stratified case-control analysis for hereditary ataxia in Parson Russell Terriers. The SNP-ID, the position on dog chromosome (CFA) in base pairs (bp) according to the dog genome assembly CanFam2, minor allele, minor allele frequency (MAF) for all, affected (MAF_A_) and unaffected (MAF_U_) dogs (controls), variance explained by the single SNP (V_SNP_), -log_10_P-values (-log_10_P) and Bonferroni-corrected -log_10_P-values (-log_10_P_Bonf_) of the general model analysis are given. Odds ratios (OR) with 95% confidence intervals (CI) are from a sex-stratified case-control analysis for cases and controls.

| CFA | Position | SNP-ID | Minor | MAF | MAF_A_ | MAF_U_ | V_SNP_ | OR | CI-L | CI-U | -log_10_P | -log_10_P_Bonf_ |
| --- | --- | --- | --- | --- | --- | --- | --- | --- | --- | --- | --- | --- |
|  |  |  | allele |  |  |  |  |  |  |  |  |  |
| 38 | 18,533,865 | BICF2G63072533 | T | 0.38 | 0.54 | 0.30 | 0.56 | 3.24 | 1.17 | 8.98 | 6.62 | 1.51 |
| 38 | 25,208,028 | BICF2P378938 | A | 0.49 | 0.83 | 0.31 | 0.53 | 0.08 | 0.02 | 0.26 | 6.55 | 1.44 |
| 38 | 25,313,048 | BICF2P478530 | T | 0.45 | 0.79 | 0.29 | 0.63 | 11.98 | 3.56 | 40.33 | 8.29 | 3.18 |
