# Supplementary material for: Genome-wide association study for hereditary ataxia in the Parson Russell Terrier and DNA-testing for ataxia-associated mutations in the Parson and Jack Russell Terrier
Source: BMC Vet Res. 2016 Oct 10;12:225. doi: 10.1186/s12917-016-0862-x (PMC5057501; doi:10.1186/s12917-016-0862-x)
Supplement: Additional file 9: — Overview on the results of extended homozygosity mapping in Parson Russell Terriers (PRT) genotyped on the canine Illumina high density beadchip. The IDs of PRT, KCNJ10 c.627C > G genotype, the SNP-IDs and positions in base pairs (bp) for the start and end of the homozygosity region are given. The PRT-ID 2 indicates dogs which were used for sequencing in this study and PRT-ID 3 indicates the designation for PRT (P.n.) which are shown in the pedigree (see Additional file 12). KCNJ10 is located at 25,148,668–25,149,816 on CFA38 (CanFam2). (DOC 51 kb) [file 12917_2016_862_MOESM9_ESM.doc]

**Additional file 9:** Overview on the results of extended homozygosity mapping in Parson Russell Terriers (PRT) genotyped on the canine Illumina high density beadchip. The IDs of PRT, *KCNJ10* c.627C>G genotype, the SNP-IDs and positions in base pairs (bp) for the start and end of the homozygosity region are given. The PRT-ID 2 indicates dogs which were used for sequencing in this study and PRT-ID 3 indicates the designation for PRT (P.n.) which are shown in the pedigree (see Additional file 14). *KCNJ10* is located at 25,148,668-25,149,816 on CFA38 (CanFam2).

| PRT-ID 1 | PRT-ID 2 | PRT-ID 3 | Genotype | SNP-ID | | Position in bp | | Size (kb) |
| --- | --- | --- | --- | --- | --- | --- | --- | --- |
|  |  |  | c.627C>G | Start | End | Start | End |  |
| TIHODOG-1 |  | P.n. 17 | G/G | BICF2P386179 | BICF2G63066776 | 25156328 | 25771992 | 615.664 |
| TIHODOG-2 | PRT II | P.n. 16 | G/G | BICF2P386179 | BICF2G63066902 | 25156328 | 25703917 | 547.589 |
| TIHODOG-3 |  | P.n. 15 | G/G | BICF2S23327387 | BICF2G63067452 | 23387952 | 25280713 | 1892.76 |
| TIHODOG-4 | PRT III |  | G/G | BICF2G63069968 | BICF2P528139 | 20919253 | 25507757 | 4588.5 |
| TIHODOG-5 | - |  | G/G | BICF2G63073040 | TIGRP2P424618_RS8457687 | 17434518 | 26894819 | 9460.3 |
| TIHODOG-6 | - |  | G/G | BICF2G63073040 | BICF2G63067240 | 17434518 | 25461376 | 8026.8 |
| TIHODOG-7 | - |  | G/G | BICF2P1027299 | TIGRP2P424618_RS8457687 | 15411810 | 26894819 | 11483 |
| TIHODOG-8 | - |  | G/G | BICF2P1279937 | BICF2G63067452 | 14943189 | 25280713 | 10337.5 |
| TIHODOG-9 | - |  | G/G | BICF2S2327071 | BICF2G63065893 | 14805311 | 26413388 | 11608.1 |
| TIHODOG-10 | - |  | G/G | BICF2P1411908 | BICF2G63067620 | 14463783 | 25118803 | 10655 |
| TIHODOG-11 | - |  | G/G | BICF2S23126165 | TIGRP2P424618_RS8457687 | 3017351 | 26894819 | 23877.5 |
| TIHODOG-12 | PRT I | P.n. 14 | C/C | BICF2G63071367 | TIGRP2P424618_RS8457687 | 19565965 | 26894819 | 7328.85 |
| Consensus region |  |  |  | BICF2P386179 | BICF2G63067452 | 25156328 | 25280713 | 124.385 |
