# Supplementary material for: Genome-wide association study for hereditary ataxia in the Parson Russell Terrier and DNA-testing for ataxia-associated mutations in the Parson and Jack Russell Terrier
Source: BMC Vet Res. 2016 Oct 10;12:225. doi: 10.1186/s12917-016-0862-x (PMC5057501; doi:10.1186/s12917-016-0862-x)
Supplement: Additional file 10: — Distribution of genotyping results for the mutation CAPN1:c.344G > A and KCNJ10:c.627C > G in different dog breeds. Numbers of tested homozygous wild-type (wt/wt), heterozygous (wt/mut) and homozygous mutant (mut/mut) genotypes are given. (DOC 51 kb) [file 12917_2016_862_MOESM10_ESM.doc]

**Additional file 10:** Distribution of genotyping results for the mutation *CAPN1:*c.344G>A and *KCNJ10:*c.627C>G in different dog breeds. Number of tested homozygous wild-type (wt/wt), heterozygous (wt/mut) and homozygous mutant (mut/mut) genotype is given.

| Breed | n | *CAPN1:*c.344G>A and *KCNJ10:*c.627C>G | | |
| --- | --- | --- | --- | --- |
|  |  | wt/wt | wt/mut | mut/mut |
| Afghan Hound | 6 | 6 | 0 | 0 |
| Akita | 16 | 16 | 0 | 0 |
| American Bulldog | 16 | 16 | 0 | 0 |
| Australian Shepherd | 13 | 13 | 0 | 0 |
| Barzoi | 5 | 5 | 0 | 0 |
| Bernese Mountain Dog | 16 | 16 | 0 | 0 |
| Boxer | 16 | 16 | 0 | 0 |
| Deerhound | 1 | 1 | 0 | 0 |
| Do Khyi | 13 | 13 | 0 | 0 |
| Elo | 16 | 16 | 0 | 0 |
| French Bulldog | 3 | 3 | 0 | 0 |
| German Drahthaar | 1 | 1 | 0 | 0 |
| Hanoverian Scenthound | 2 | 2 | 0 | 0 |
| Kuvasz | 1 | 1 | 0 | 0 |
| Polski Owczarek Nizinny | 1 | 1 | 0 | 0 |
| Rhodesian Ridgeback | 1 | 1 | 0 | 0 |
| Samoyed | 7 | 7 | 0 | 0 |
| Siberian Husky | 18 | 18 | 0 | 0 |
| Tibetan Terrier | 24 | 24 | 0 | 0 |
| Yorkshire Terrier | 3 | 3 | 0 | 0 |
| Total | 179 | 179 | 0 | 0 |
