# Supplementary material for: Genome-wide association study for hereditary ataxia in the Parson Russell Terrier and DNA-testing for ataxia-associated mutations in the Parson and Jack Russell Terrier
Source: BMC Vet Res. 2016 Oct 10;12:225. doi: 10.1186/s12917-016-0862-x (PMC5057501; doi:10.1186/s12917-016-0862-x)
Supplement: Additional file 11: — Identification of variants in the gene KCNJ10 in affected and unaffected Parson Russell Terriers (PRT) and Jack Russell Terriers (JRT). IDs of the variants, locations in the gene, accession numbers and the genotype for each variant are given. The single nucleotide variant reported by Gilliam et al. [7] (KCNJ10:c.627C > G) written in bold. Indel mutations are denoted as wt (wild-type) or mut (mutant). (DOC 108 kb) [file 12917_2016_862_MOESM11_ESM.doc]

**Additional file 11:** Identification of variants in the gene *KCNJ10* in affected and unaffected Parson Russell Terriers (PRT) and Jack Russell Terriers (JRT). IDs of the variants, locations in the gene, accession numbers and the genotype for each variant are given. The single nucleotide variant reported by Gilliam et al. (2014) (*KCNJ10:*c.627C>G) [7] written in bold. Indel mutations are denoted as wt (wild-type) or mut (mutant).

|  |  |  | Phenotypes | | | | | | | | | | | | |
| --- | --- | --- | --- | --- | --- | --- | --- | --- | --- | --- | --- | --- | --- | --- | --- |
|  |  |  | Hereditary ataxia (clinically and histopathologically diagnosed) | | | Suspected hereditary ataxia  (clinical signs of ataxia, reported by the veterinarian or owner) | | | | | unaffected dogs | | Suspected hereditary ataxia (clinical signs of ataxia, reported by the veterinarian) | | |
| ID | Location in gene (genomic level) | Number of PCR-Amplicon | PRT I  P.n. 14 | PRT II  P.n. 16 | PRT III | PRT IV | PRT V | PRT VI | PRT VII | PRT VIII | PRT IV | PRT X | JRT I | JRT II | JRT III |
| g.22130346A>G | intron 1 (5`UTR) | 2 | A/A | A/G | A/A | A/A | A/A | A/A | n.s. | n.s. | A/A | n.s. | n.s. | n.s. | n.s. |
| g.22130381G>A | intron 1 (5`UTR) | 2 | A/G | G/G | G/G | G/G | G/G | G/G | n.s. | n.s. | G/G | n.s. | n.s. | n.s. | n.s. |
| g.22130576G>A | exon 2 (5`UTR) | 2 | A/G | G/G | G/G | A/G | G/G | G/G | n.s. | n.s. | A/G | n.s. | n.s. | n.s. | n.s. |
| g.22130585C>G | exon 2 | 2 | C/G | C/C | C/C | C/G | C/C | C/C | n.s. | n.s. | C/G | n.s. | n.s. | n.s. | n.s. |
| g.22130747T>C | intron 2 | 2 | C/C | T/T | T/T | C/C | T/T | T/T | n.s. | n.s. | T/T | n.s. | n.s. | n.s. | n.s. |
| g.22130774A>G | intron 2 | 2 | A/G | A/A | A/A | A/G | A/A | A/A | n.s. | n.s. | A/A | n.s. | n.s. | n.s. | n.s. |
| g.22139600T>G | intron 2 | 3.1 | G/G | G/G | G/G | G/G | G/G | G/G | n.s. | n.s. | G/G | n.s. | n.s. | n.s. | n.s. |
| g.22139606C>T | intron 2 | 3.1 | T/T | T/T | T/T | T/T | T/T | T/T | n.s. | n.s. | T/T | n.s. | n.s. | n.s. | n.s. |
| g.22139775C>T | exon 3 | 3.1 | C/T | T/T | T/T | C/C | T/T | T/T | n.s. | n.s. | T/T | n.s. | n.s. | n.s. | n.s. |
| **g.22140300C>G** | **exon 3** | **3.1** | **C/C** | **G/G** | **G/G** | **C/C** | **G/G** | **G/G** | **G/G** | **C/C** | **C/G** | **C/C** | **G/G** | **C/G** | **C/C** |
| g.22140866C>T | exon 3  (3`UTR) | 3.2 | C/C | C/C | C/C | C/T | C/C | C/C | C/C | C/C | C/C | C/C | C/C | C/C | C/T |
| g.22141027insC | exon 3  (3`UTR) | 3.2 | wt/mut | mut/mut | mut/mut | wt/wt | mut/mut | mut/mut | mut/mut | wt/wt | mut/mut | wt/wt | mut/mut | mut/mut | wt/wt |
| g.22141093T>G | exon 3  (3`UTR) | 3.2 | T/T | G/T | T/T | T/T | T/T | T/T | T/T | n.d. | T/T | T/T | T/T | T/T | T/T |
| g.22141136delC | exon 3  (3`UTR) | 3.3 | mut/mut | mut/mut | n.d. | n.d. | mut/mut | mut/mut | n.d. | mut/mut | n.d. | mut/mut | mut/mut | mut/mut | mut/mut |
| g.22141273C>A | exon 3  (3`UTR) | 3.3 | C/C | C/C | n.d. | n.d. | C/C | C/C | C/C | C/C | n.d. | C/A | C/C | C/C | C/C |
| g.22141310C>T | exon 3  (3`UTR) | 3.3 | C/T | T/T | n.d. | n.d. | T/T | T/T | T/T | C/C | n.d. | C/C | T/T | T/T | C/C |
| g.22141588C>T | exon 3  (3`UTR) | 3.3 | C/C | C/C | n.d. | n.d. | C/C | C/C | C/C | T/T | n.d. | C/C | C/C | C/C | C/C |
| g.22141762G>A | exon 3  (3`UTR) | 3.3 | G/G | G/G | n.d. | n.d. | G/G | G/G | G/G | A/A | n.d. | G/G | G/G | G/G | G/G |
| g.22141781C>G | exon 3  (3`UTR) | 3.3 | C/C | G/G | n.d. | n.d. | C/C | C/C | G/G | C/C | n.d. | G/G | G/G | G/G | G/G |
| g.22141869C>T | exon 3  (3`UTR) | 3.3 | C/C | C/C | C/C | n.d. | C/C | C/C | T/T | C/C | n.d. | C/C | T/T | T/T | C/T |
| g.22142363C>T | exon 3  (3`UTR) | 3.4 | C/C | T/T | T/T | C/C | T/T | T/T | n.d. | n.d. | T/T | n.d. | n.d. | n.d. | n.d. |
| g.22142532insGAGCGC | exon 3  (3`UTR) | 3.5 | mut/mut | mut/mut | mut/mut | n.d. | mut/mut | mut/mut | mut/mut | mut/mut | mut/mut | mut/mut | mut/mut | mut/mut | n.d. |
| g.22142532T>C | exon 3  (3`UTR) | 3.5 | C/T | C/C | C/C | n.d. | C/C | C/C | C/C | C/C | C/C | C/C | C/C | C/C | n.d. |
| g.22142545A>G | exon 3  (3`UTR) | 3.5 | A/A | A/A | A/G | n.d. | A/A | A/A | A/A | A/G | A/A | A/A | A/A | A/A | n.d. |
| g.22142547C>T | exon 3  (3`UTR) | 3.5 | C/C | C/C | C/C | n.d. | C/C | C/C | C/C | C/T | C/C | C/C | C/T | C/C | n.d. |
| g.22142548G>A | exon 3  (3`UTR) | 3.5 | G/G | G/G | G/G | n.d. | G/G | G/G | G/G | A/G | G/G | G/G | A/G | G/G | n.d. |
| g.22142581C>A | exon 3  (3`UTR) | 3.5 | C/C | C/C | C/C | C/C | C/C | C/C | C/C | A/C | C/C | C/C | C/C | C/C | n.d. |
| g.22142600G>A | exon 3  (3`UTR) | 3.5 | A/G | G/G | G/G | A/G | G/G | G/G | G/G | A/A | G/G | G/G | G/G | G/G | n.d. |
| g.22142727T>A | exon 3  (3`UTR) | 3.5 | T/T | T/T | T/T | T/T | T/T | T/T | T/T | T/T | T/T | T/T | A/T | A/T | n.d. |
| g.22143065G>A | exon 3  (3`UTR) | 3.5 | G/G | G/G | A/G | n.d. | A/G | G/G | G/G | G/G | G/G | G/G | G/G | G/G | n.d. |
| g.22143082C>T | exon 3  (3`UTR) | 3.5 | C/T | T/T | T/T | n.d. | T/T | T/T | T/T | C/C | T/T | C/T | T/T | T/T | n.d. |
| g.22143100C>T | exon 3  (3`UTR) | 3.5 | C/T | T/T | T/T | n.d. | T/T | T/T | T/T | C/C | T/T | C/T | T/T | T/T | n.d. |
| g.22143153G>A | exon 3  (3`UTR) | 3.5 | A/G | A/G | A/A | G/G | A/A | A/A | A/A | G/G | A/A | A/A | A/A | A/A | n.d. |
| g.22143184A>G | exon 3  (3`UTR) | 3.6 | A/A | A/G | A/G | A/A | n.d. | A/A | n.s. | n.s. | n.d. | n.s. | n.s. | n.s. | n.s. |
| g.22143220A>G | exon 3  (3`UTR) | 3.6 | A/A | A/G | A/G | A/G | n.d. | A/A | n.s. | n.s. | n.d. | n.s. | n.s. | n.s. | n.s. |
| g.22143251T>C | exon 3  (3`UTR) | 3.6 | C/T | C/C | C/C | C/T | n.d. | C/C | n.s. | n.s. | n.d. | n.s. | n.s. | n.s. | n.s. |
| g.22143252G>T | exon 3  (3`UTR) | 3.6 | G/T | T/T | T/T | G/T | n.d. | T/T | n.s. | n.s. | n.d. | n.s. | n.s. | n.s. | n.s. |
| g.22143279C>A | exon 3  (3`UTR) | 3.6 | C/C | A/C | C/C | C/C | n.d. | C/C | n.s. | n.s. | n.d. | n.s. | n.s. | n.s. | n.s. |
| g.22143282G>A | exon 3  (3`UTR) | 3.6 | G/G | A/G | A/G | G/G | n.d. | G/G | n.s. | n.s. | n.d. | n.s. | n.s. | n.s. | n.s. |
| g.22143340G>A | exon 3  (3`UTR) | 3.6 | G/G | A/A | A/A | G/G | n.d. | A/A | n.s. | n.s. | n.d. | n.s. | n.s. | n.s. | n.s. |
| g.22143562A>G | exon 3  (3`UTR) | 3.6 | A/A | A/G | A/G | A/A | n.d. | A/A | n.s. | n.s. | n.d. | n.s. | n.s. | n.s. | n.s. |
| g.22143582insTTTGGT | exon 3  (3`UTR) | 3.6 | wt/wt | mut/mut | mut/mut | wt/wt | n.d. | mut/mut | n.s. | n.s. | n.d. | n.s. | n.s. | n.s. | n.s. |
| g.22143666C>A | 3` d.s. | 3.6 | C/C | A/A | A/A | C/C | n.d. | A/A | n.s. | n.s. | n.d. | n.s. | n.s. | n.s. | n.s. |
| g.22143685C>T | 3` d.s. | 3.6 | C/C | T/T | C/T | C/C | n.d. | T/T | n.s. | n.s. | n.d. | n.s. | n.s. | n.s. | n.s. |
| g.22143818C>T | 3` d.s. | 3.6 | C/T | C/C | n.d. | T/T | n.d. | C/C | n.s. | n.s. | n.d. | n.s. | n.s. | n.s. | n.s. |

P.n: Pedigree number; wt: wildtype; mut: mutant; d.s.: downstream sequence; n.d.: not determined; n.s.: not sequenced
