# Supplementary material for: Genome-wide association study for hereditary ataxia in the Parson Russell Terrier and DNA-testing for ataxia-associated mutations in the Parson and Jack Russell Terrier
Source: BMC Vet Res. 2016 Oct 10;12:225. doi: 10.1186/s12917-016-0862-x (PMC5057501; doi:10.1186/s12917-016-0862-x)
Supplement: Additional file 12: — Sequencing results of nine dogs of different breeds for evaluation of the variants detected in Parson Russell Terriers (PRT) and Jack Russell Terriers (JRT). IDs of the variants and the genotype for each variant are given. The single nucleotide variant reported by Gilliam et al. [7] (KCNJ10:c.627C > G) written in bold. Indel mutations are denoted as wt (wildtype) or mut (mutant). Indel mutations are denoted as wt (wild-type) or mut (mutant). (DOC 97 kb) [file 12917_2016_862_MOESM12_ESM.doc]

**Additional file 12:** Sequencing results of nine dogs of different breeds for evaluation of the variants detected in Parson Russell Terriers (PRT) and Jack Russell Terriers (JRT). IDs of the variants and the genotype for each variant are given. The single nucleotide variant reported by Gilliam et al. (2014) (*KCNJ10:*c.627C>G) [7] written in bold. Indel mutations are denoted as wt (wild-type) or mut (mutant).

| ID | Akita | Bernese mountain dog | Dalmatian | German Drahthaar | German shepherd | Irish wolfhound | Shar Pei | Tibetan terrier | Briard |
| --- | --- | --- | --- | --- | --- | --- | --- | --- | --- |
| g.22130346A>G | A/A | A/A | A/A | A/A | A/A | A/A | A/A | A/A | A/A |
| g.22130381G>A | G/G | G/G | G/G | G/G | A/A | G/G | G/G | G/G | G/G |
| g.22130576G>A | G/G | A/G | A/G | A/G | G/G | G/G | A/G | G/G | G/G |
| g.22130585C>G | C/C | C/G | C/G | C/G | C/C | C/C | C/G | C/C | C/C |
| g.22130747T>C | C/C | C/C | C/C | C/C | C/C | C/C | C/T | C/T | T/T |
| g.22130774A>G | G/G | G/G | G/G | A/G | G/G | G/G | A/G | A/G | A/A |
| g.22139600T>G | T/T | G/G | G/T | G/T | T/T | G/G | G/T | G/G | G/G |
| g.22139606C>T | C/C | T/T | C/T | C/T | C/C | T/T | C/T | T/T | T/T |
| g.22139775C>T | C/C | C/C | C/C | C/T | C/C | C/C | C/C | C/T | C/C |
| **g.22140300C>G** | **C/C** | **C/C** | **C/C** | **C/C** | **C/C** | **C/C** | **C/C** | **C/C** | **C/C** |
| g.22140866C>T | C/C | C/C | C/T | C/C | C/C | C/C | C/T | C/C | C/C |
| g.22141027insC | wt/wt | wt/wt | wt/wt | wt/wt | wt/wt | wt/wt | wt/wt | wt/wt | wt/wt |
| g.22141093T>G | T/T | T/T | T/T | T/T | T/T | T/T | T/T | T/T | T/T |
| g.22141136delC | ./. | mut/mut | mut/mut | mut/mut | wt/mut | wt/wt | wt/mut | wt/mut | mut/mut |
| g.22141273C>A | C/C | C/C | C/C | C/C | C/C | C/C | C/C | C/C | C/C |
| g.22141310C>T | C/C | C/C | C/C | C/T | C/C | C/C | C/C | C/T | C/C |
| g.22141588C>T | T/T | C/T | C/C | C/T | C/T | C/C | C/C | C/C | C/T |
| g.22141762G>A | G/G | A/G | G/G | G/G | A/G | G/G | G/G | G/G | A/G |
| g.22141781C>G | G/G | C/G | C/C | C/G | C/G | C/C | C/C | C/C | C/G |
| g.22141869C>T | C/C | C/C | C/T | C/T | C/C | C/C | C/T | C/T | C/C |
| g.22142363C>T | C/C | C/C | C/C | C/C | C/C | C/C | C/C | C/T | C/C |
| g.22142532insGAGCGC | wt/mut | wt/mut | wt/mut | mut/mut | wt/mut | wt/wt | wt/mut | wt/mut | wt/mut |
| g.22142532T>C | T/T | T/T | T/T | C/T | T/T | T/T | T/T | T/T | T/T |
| g.22142545A>G | A/A | A/A | A/A | A/A | A/A | A/A | A/A | A/A | A/A |
| g.22142547C>T | C/C | C/C | C/C | C/C | C/C | C/C | C/C | C/C | C/C |
| g.22142548G>A | G/G | G/G | G/G | G/G | G/G | G/G | G/G | G/G | G/G |
| g.22142581C>A | C/C | C/C | C/C | C/C | C/C | C/C | C/C | C/C | C/C |
| g.22142600G>A | G/G | A/G | A/G | G/G | A/G | G/G | A/G | G/G | G/G |
| g.22142727T>A | T/T | T/T | T/T | T/T | T/T | T/T | T/T | T/T | T/T |
| g.22143065G>A | G/G | G/G | G/G | G/G | G/G | G/G | G/G | G/G | G/G |
| g.22143082C>T | C/C | C/C | C/T | C/T | C/C | C/C | C/C | C/T | C/C |
| g.22143100C>T | C/C | C/C | C/T | C/T | C/C | C/C | C/C | C/T | C/C |
| g.22143153G>A | G/G | G/G | A/G | A/A | G/G | G/G | G/G | A/G | G/G |
| g.22143184A>G | A/A | A/A | A/A | A/A | A/A | A/A | A/A | A/A | A/A |
| g.22143220A>G | A/A | A/A | A/A | A/A | A/A | A/A | A/A | A/A | A/A |
| g.22143251T>C | C/C | C/T | C/T | C/C | C/T | C/C | C/T | C/C | C/C |
| g.22143252G>T | T/T | G/T | G/T | T/T | G/T | T/T | G/T | T/T | T/T |
| g.22143279C>A | C/C | C/C | C/C | C/C | C/C | C/C | C/C | C/C | C/C |
| g.22143282G>A | G/G | G/G | G/G | G/G | G/G | G/G | G/G | G/G | G/G |
| g.22143340G>A | G/G | G/G | G/G | G/G | G/G | G/G | G/G | A/G | G/G |
| g.22143562A>G | A/A | A/A | A/A | A/A | A/A | A/A | A/A | A/A | A/A |
| g.22143582insTTTGGT | mut/mut | wt/wt | wt/mut | wt/mut | wt/wt | wt/wt | wt/mut | wt/mut | wt/wt |
| g.22143666C>A | C/C | C/C | A/C | C/C | C/C | C/C | C/C | A/C | C/C |
| g.22143685C>T | T/T | C/C | C/T | C/C | C/C | C/C | C/T | C/T | C/C |
| g.22143818C>T | C/C | C/T | C/T | C/C | T/T | C/C | C/T | C/C | C/C |

wt: wild-type; mut: mutant; ./.: not determined
