# Supplementary material for: Genome-wide association study for hereditary ataxia in the Parson Russell Terrier and DNA-testing for ataxia-associated mutations in the Parson and Jack Russell Terrier
Source: BMC Vet Res. 2016 Oct 10;12:225. doi: 10.1186/s12917-016-0862-x (PMC5057501; doi:10.1186/s12917-016-0862-x)
Supplement: Additional file 13: — Variants in the gene KCNJ10 which are private for Parson Russell Terriers (PRT) and Jack Russell Terriers (JRT). IDs of the variants, locations in the gene, accession numbers and the genotype for each variant are given. Both the single nucleotide variant reported by Gilliam et al. [7] (KCNJ10:c.627C > G) and KCNJ10:g.22141027insC identified as further putative variant written in bold. Indel mutations are denoted as wt (wild-type) or mut (mutant). (DOC 64 kb) [file 12917_2016_862_MOESM13_ESM.doc]

**Additional file 13:** Variants in the gene *KCNJ10* which are private for Parson Russell Terriers (PRT) and Jack Russell Terriers (JRT). IDs of the variants, locations in the gene, accession numbers and the genotype for each variant are given. Both the single nucleotide variant reported by Gilliam et al. (2014) (*KCNJ10:*c.627C>G) [7] and *KCNJ10:*g.22141027insC identified as further putative variant written in bold. Indel mutations are denoted as wt (wild-type) or mut (mutant).

|  |  |  | Phenotypes | | | | | | | | | | | | |
| --- | --- | --- | --- | --- | --- | --- | --- | --- | --- | --- | --- | --- | --- | --- | --- |
|  |  |  | hereditary ataxia (clinically and histopathologically diagnosed) | | | hereditary ataxia  (clinical signs of ataxia, reported by the veterinarian and owner) | | | | | unaffected dogs | | hereditary ataxia (clinical signs of ataxia, reported by the veterinarian) | | |
| ID | Location in gene (genomic level) | Number of PCR-Amplicon | PRT I  P.n. 14 | PRT II  P.n. 16 | PRT III | PRT IV | PRT V | PRT VI | PRT VII | PRT VIII | PRT IV | PRT X | JRT I | JRT II | JRT III |
| g.22130346A>G | intron 1 (5`UTR) | 2 | A/A | A/G | A/A | A/A | A/A | A/A | n.s. | n.s. | A/A | n.s. | n.s. | n.s. | n.s. |
| **g.22140300C>G** | **exon 3** | **3.1** | **C/C** | **G/G** | **G/G** | **C/C** | **G/G** | **G/G** | **G/G** | **C/C** | **C/G** | **C/C** | **G/G** | **C/G** | **C/C** |
| **g.22141027insC** | **exon 3 (3`UTR)** | **3.2** | **wt/mut** | **mut/mut** | **mut/mut** | **wt/wt** | **mut/mut** | **mut/mut** | **mut/mut** | **wt/wt** | **mut/mut** | **wt/wt** | **mut/mut** | **mut/mut** | **wt/wt** |
| g.22141093T>G | exon 3 (3`UTR) | 3.2 | T/T | G/T | T/T | T/T | T/T | T/T | T/T | n.d. | T/T | T/T | T/T | T/T | T/T |
| g.22141273C>A | exon 3 (3`UTR) | 3.3 | C/C | C/C | n.d. | n.d. | C/C | C/C | C/C | C/C | n.d. | C/A | C/C | C/C | C/C |
| g.22142545A>G | exon 3 (3`UTR) | 3.5 | A/A | A/A | A/G | n.d. | A/A | A/A | A/A | A/G | A/A | A/A | A/A | A/A | n.d. |
| g.22142547C>T | exon 3 (3`UTR) | 3.5 | C/C | C/C | C/C | n.d. | C/C | C/C | C/C | C/T | C/C | C/C | C/T | C/C | n.d. |
| g.22142548G>A | exon 3 (3`UTR) | 3.5 | G/G | G/G | G/G | n.d. | G/G | G/G | G/G | A/G | G/G | G/G | A/G | G/G | n.d. |
| g.22142581C>A | exon 3 (3`UTR) | 3.5 | C/C | C/C | C/C | C/C | C/C | C/C | C/C | A/C | C/C | C/C | C/C | C/C | n.d. |
| g.22142727T>A | exon 3 (3`UTR) | 3.5 | T/T | T/T | T/T | T/T | T/T | T/T | T/T | T/T | T/T | T/T | A/T | A/T | n.d. |
| g.22143065G>A | exon 3 (3`UTR) | 3.5 | G/G | G/G | A/G | n.d. | A/G | G/G | G/G | G/G | G/G | G/G | G/G | G/G | n.d. |
| g.22143184A>G | exon 3 (3`UTR) | 3.6 | A/A | A/G | A/G | A/A | n.d. | A/A | n.s. | n.s. | n.d. | n.s. | n.s. | n.s. | n.s. |
| g.22143220A>G | exon 3 (3`UTR) | 3.6 | A/A | A/G | A/G | A/G | n.d. | A/A | n.s. | n.s. | n.d. | n.s. | n.s. | n.s. | n.s. |
| g.22143279C>A | exon 3 (3`UTR) | 3.6 | C/C | A/C | C/C | C/C | n.d. | C/C | n.s. | n.s. | n.d. | n.s. | n.s. | n.s. | n.s. |
| g.22143282G>A | exon 3 (3`UTR) | 3.6 | G/G | A/G | A/G | G/G | n.d. | G/G | n.s. | n.s. | n.d. | n.s. | n.s. | n.s. | n.s. |
| g.22143562A>G | exon 3 (3`UTR) | 3.6 | A/A | A/G | A/G | A/A | n.d. | A/A | n.s. | n.s. | n.d. | n.s. | n.s. | n.s. | n.s. |

P.n: Pedigree number; wt: wild-type; mut: mutant; n.d.: not determined; n.s.: not sequenced
