# Supplementary material for: Genome-wide association study for hereditary ataxia in the Parson Russell Terrier and DNA-testing for ataxia-associated mutations in the Parson and Jack Russell Terrier
Source: BMC Vet Res. 2016 Oct 10;12:225. doi: 10.1186/s12917-016-0862-x (PMC5057501; doi:10.1186/s12917-016-0862-x)

Additional file 14: The pedigree demonstrates the relationship of 17 Parson Russell Terriers (PRT) (Pedigree numbers 1-17) selected from the sample of our study. Genotypes for both, the *KNCJ10:*g.22141027insC (first row) and *KCNJ10:*c.627C>G (Gilliam et al., 2014) [7] (second row) variant are given for each of the 17 dogs. *KNCJ10:*g.22141027insC genotyping resulted in 5/17 wild-type (wt/wt), 9/17 heterozygous (wt/mut) and 3/17 homozygous mutant (mut/mut) PRT. *KCNJ10:*c.627C>G genotyping resulted in 9/17 wild-type (wt/wt), 5/17 heterozygous (wt/mut) and 3/17 homozygous mutant (mut/mut) PRT.

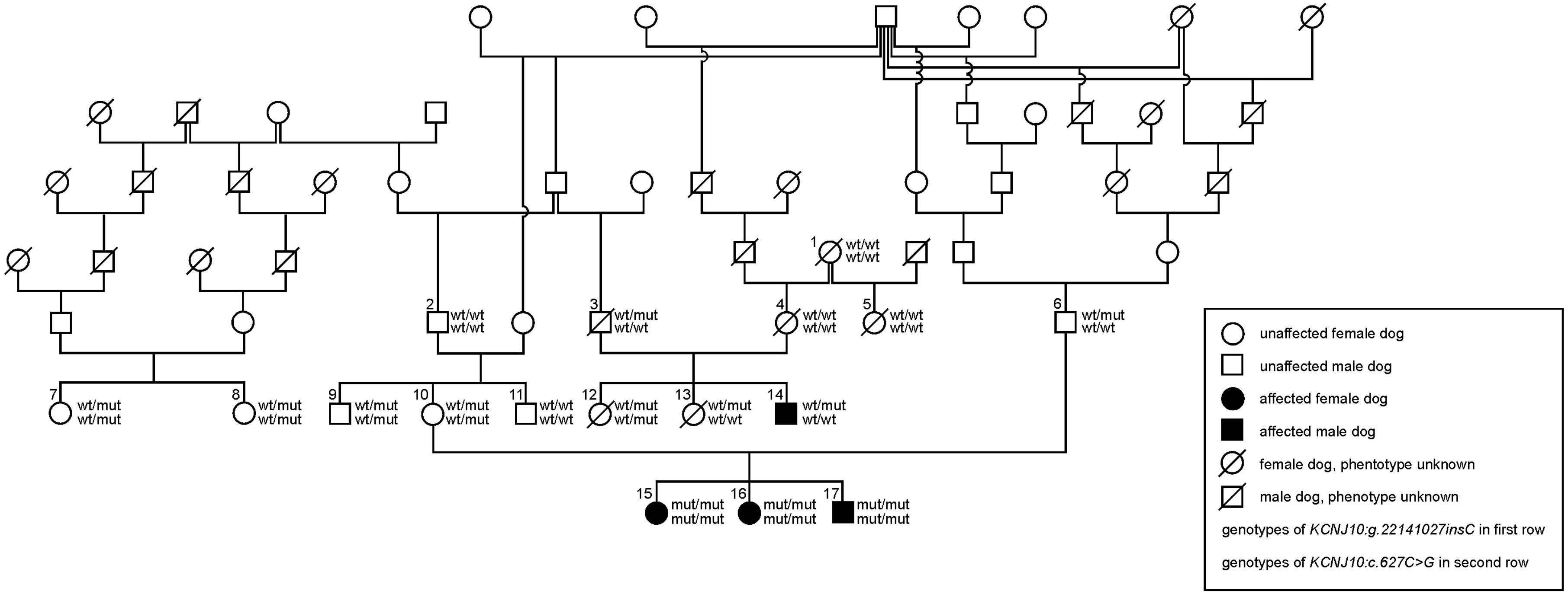

Supplement: Additional file 14: — The pedigree demonstrates the relationship of 17 Parson Russell Terriers (PRT) (pedigree number 1–17) selected from the sample of our study. Genotypes for both, the KNCJ10:g.22141027insC (first row) and KCNJ10:c.627C > G [7] (second row) variant are given for each of the 17 dogs. KNCJ10:g.22141027insC genotyping resulted in 5/17 wild-type (wt/wt), 9/17 heterozygous (wt/mut) and 3/17 homozygous mutant (mut/mut) PRT. KCNJ10:c.627C > G genotyping resulted in 9/17 wild-type (wt/wt), 5/17 heterozygous (wt/mut) and 3/17 homozygous mutant (mut/mut) PRT. (PDF 22 kb) [file 12917_2016_862_MOESM14_ESM.pdf]
