# Supplementary material for: Genome-wide association study for hereditary ataxia in the Parson Russell Terrier and DNA-testing for ataxia-associated mutations in the Parson and Jack Russell Terrier
Source: BMC Vet Res. 2016 Oct 10;12:225. doi: 10.1186/s12917-016-0862-x (PMC5057501; doi:10.1186/s12917-016-0862-x)
Supplement: Additional file 15: — Distribution of genotyping results for the mutation KCNJ10:g.22141027insC in different dog breeds. Numbers of tested homozygous wild-type (wt/wt), heterozygous (wt/mut) and homozygous mutant (mut/mut) genotypes are given. (DOC 57 kb) [file 12917_2016_862_MOESM15_ESM.doc]

**Additional file 15:** Distribution of genotyping results for the mutation *KCNJ10:*g.22141027insC in different dog breeds. Number of tested homozygous wild-type (wt/wt), heterozygous (wt/mut) and homozygous mutant (mut/mut) genotype is given.

| Breed |  | *KCNJ10:*g.22141027insC | | |
| --- | --- | --- | --- | --- |
|  | n | wt/wt | wt/mut | mut/mut |
| Afghan Hound | 7 | 1 | 4 | 2 |
| Akita Inu | 4 | 4 | 0 | 0 |
| Alaskan Malamute | 2 | 2 | 0 | 0 |
| American Bulldog | 1 | 1 | 0 | 0 |
| American Staffordshire | 1 | 1 | 0 | 0 |
| Australian Shepherd | 2 | 0 | 2 | 0 |
| Barzoi | 5 | 0 | 3 | 2 |
| Beagle | 2 | 2 | 0 | 0 |
| Belgian Shepherd | 1 | 1 | 0 | 0 |
| Bernese Mountain dog | 1 | 1 | 0 | 0 |
| Border Collie | 1 | 1 | 0 | 0 |
| Briard | 2 | 2 | 0 | 0 |
| Dachshund | 2 | 2 | 0 | 0 |
| Deerhound | 1 | 0 | 1 | 0 |
| Do Khyi | 1 | 1 | 0 | 0 |
| English Cocker Spaniel | 1 | 1 | 0 | 0 |
| English Springer Spaniel | 1 | 1 | 0 | 0 |
| Entlebucher Mountain dog | 1 | 1 | 0 | 0 |
| French Bulldog | 5 | 2 | 3 | 0 |
| German Shepherd | 2 | 2 | 0 | 0 |
| German Drahthaar | 1 | 0 | 1 | 0 |
| Golden Retriever | 1 | 1 | 0 | 0 |
| Great Dane | 3 | 3 | 0 | 0 |
| Great Pyrenees | 2 | 2 | 0 | 0 |
| Hanoverian scenthound | 2 | 0 | 2 | 0 |
| Hovawart | 2 | 2 | 0 | 0 |
| Italian Greyhound | 3 | 3 | 0 | 0 |
| Kuvasz | 3 | 2 | 1 | 0 |
| Labrador Retriever | 1 | 1 | 0 | 0 |
| Lagotto Romagnolo | 2 | 2 | 0 | 0 |
| Newfoundland | 3 | 3 | 0 | 0 |
| Polski Owczarek Nizinny | 2 | 1 | 1 | 0 |
| Rhodesian Ridgeback | 2 | 1 | 1 | 0 |
| Rough Collie | 1 | 1 | 0 | 0 |
| Saluki | 1 | 1 | 0 | 0 |
| Samoyed | 4 | 4 | 0 | 0 |
| Shiba Inu | 2 | 2 | 0 | 0 |
| Siberian Husky | 3 | 1 | 2 | 0 |
| Tibetan Terrier | 1 | 1 | 0 | 0 |
| Weimaraner | 2 | 2 | 0 | 0 |
| Total | 84 | 59 | 21 | 4 |
